# Extraction of Phenolic Compounds from the Pulp of *Myrciaria dubia* Using Eco-Friendly Solvents: Extraction Kinetics and Determination of Mass Transfer and Thermodynamic Parameters

**DOI:** 10.1021/acsomega.6c02599

**Published:** 2026-09-18

**Authors:** André Gomes Mesquita, Adriny dos Santos Miranda Lobato, Iane Valente Pires, Luiza Helena Meller da Silva, Antonio Manoel da Cruz Rodrigues

**Affiliations:** Programa de Pos-Graduacao em Ciencia e Tecnologia de Alimentos, Universidade Federal do Para, Para 66075-900, Brazil

## Abstract

Sustainable extraction of bioactive compounds from Amazonian fruits is essential for green chemistry, but integrated kinetic, thermodynamic, economic, and sustainability assessments for *Myrciaria dubia* (camu–camu) phenolics remain limited. This study investigated phenolic extraction from camu–camu pulp using natural deep eutectic solvents (NADES) composed of choline chloride and acetic acid, either pure or in binary mixtures with water or ethanol (EtOH), combined with conventional (CE), shear (SE), and ultrasound-assisted extraction (UAE; 420, 490, 560 W). Extraction kinetics, mass transfer parameters, thermodynamics, environmental performance (EcoScale and AGREE metrics), and preliminary economics were evaluated. UAE using NADES + EtOH at 560 W yielded the highest phenolic content (17.11 mg GAE/g). Fick’s diffusion model best described the kinetics (*R* ^2^ > 0.9523), indicating a diffusion-controlled process, while thermodynamic analysis confirmed an endothermic and spontaneous process. All DES-based systems showed superior environmental performance over CE using 70% (v/v) EtOH, with EcoScale scores of 87.97–92.97 (vs 83.43) and AGREE values of 0.61 (vs 0.52). Preliminary economic assessment indicated extract production costs of US$ 1.83–2.26/kg; NADES-based systems maintained competitive costs while providing higher phenolic content than conventional extraction. Combining NADES + EtOH and UAE represents a promising strategy for enhancing phenolic recovery while maintaining favorable sustainability and economic performance, supporting greener extraction of Amazonian resources.

## Graphical abstract

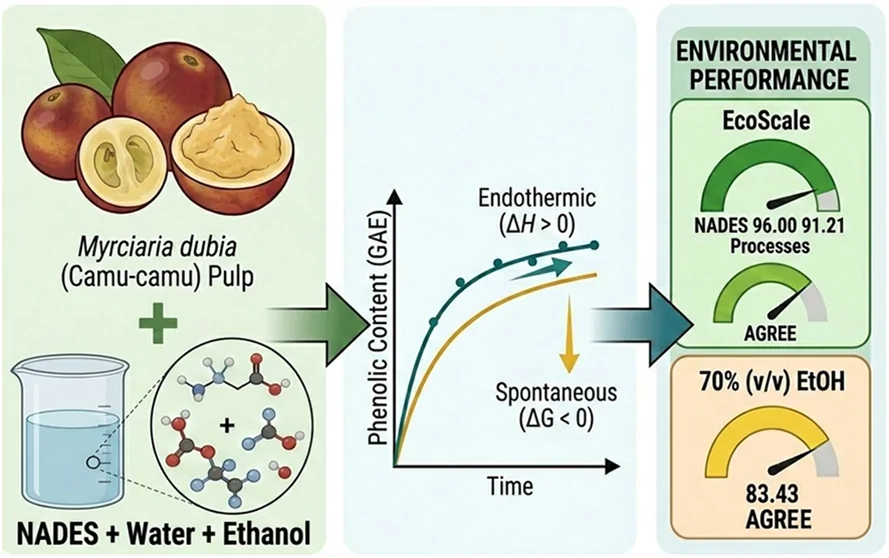

## 1 Introduction

*Myrciaria dubia*, commonly known as camu–camu, is a native species of the Amazon that has gained prominence due to the high concentration of phenolic compounds in its pulp.^1^ These bioactive molecules, recognized for their antioxidant, neuroprotective, and free radical-fighting properties, position camu–camu as a promising source for the development of nutraceutical and cosmetic products.^2^ This approach enables the replacement of synthetic ingredients with natural alternatives, the enhancement of the fruit’s added value, and the adoption of environmentally safe processes, especially in producing regions, strengthening local production chains and contributing to the sustainable use of Amazonian resources.^1^

The use of conventional extraction methods (CE), which require long processing times and high temperatures, such as Soxhlet extraction, reflux, and mechanical stirring, which require long processing times and high temperatures, has become less attractive due to high energy consumption and the risk of degradation of heat-sensitive compounds. As an alternative, innovative methods such as ultrasound-assisted extraction (UAE) and shear extraction (SE) have been improved. UAE uses ultrasonic waves to induce cavitation and rupture plant cells, releasing bioactive compounds quickly and efficiently.^3,4^ SE employs mechanical shear forces to fragment the plant matrix, accelerating mass transfer without high energy demands.^5^ The efficiency of these methods is directly related to mass transfer, a critical factor in bioactive compound extraction processes, as it determines the solute diffusion rate from the plant matrix to the solvent.^6^ Understanding and optimizing this phenomenon are essential to maximize extraction yield and ensure process selectivity, especially when using solvents with specific physicochemical characteristics, such as natural deep eutectic solvents (NADES).

Various kinetic models have been employed to understand the release dynamics of phenolic compounds from plant matrices. Their selection is based on their applicability to bioactive compound extraction processes and their ability to describe different mass transfer mechanisms.^7–10^ Among the different models used are Fick’s law of diffusion, which describes the internal transport of solutes by concentration gradient; the pseudo-second order (PSO) model, traditionally applied to adsorption but also used inversely to describe the release of compounds from the solid phase to the liquid phase;^11^ the partition model, which considers the equilibrium between solid and liquid phases;^12^ and the empirical Weibull and Peleg models, widely used for fitting nonlinear experimental data due to their mathematical flexibility and robustness.^13,14^

Sustainability, however, does not depend solely on the extraction technique, but also on the choice of solvent. In this context, eco-friendly solvents such as water, ethanol (EtOH), and NADES emerge as viable alternatives. NADES, in particular, are formed by the combination of natural substances that form hydrogen bonds, resulting in solvents with low toxicity, high stability, and adjustable properties.^15^ These solvents have already demonstrated effectiveness for different matrices such as agro-industrial byproducts of orange,^16^ olive oil,^17^ red araçá,^18^ and pitaya.^19^ Despite their environmental advantages, NADES exhibit high viscosity, which can limit the diffusion of bioactive compounds during the extraction process. To overcome this limitation, the addition of cosolvents, such as water and EtOH, has proven to be an efficient strategy for reducing viscosity without compromising the stability of the intermolecular interactions that characterize NADES.^20^

The characterization of these molecular interactions, both among NADES components and between solvent and solute, can be further explored using spectroscopic techniques such as Fourier transform infrared (FTIR) and Raman spectroscopy. These analyses allow the identification of hydrogen-bond formation and other relevant functional interactions, providing structural support for understanding the efficiency of NADES systems, whether pure or combined, in the extraction of phenolic compounds.^21^ Thus, NADES have shown great potential for application in the extraction of phenolic and bioactive compounds from various food matrices across different continents, with Asia and Europe accounting for 89% of studies using NADES.^22^ South America, despite the rich biodiversity of Brazil and the Amazon region, still has significant room for growth in NADES research. Among the countries with the highest number of publications are China, followed by Spain, Italy, India, and Brazil. In Latin America, Brazil leads in publications, followed by Colombia, Ecuador, and Peru.^22^

The literature indicates that NADES-based systems, either pure or combined with cosolvents, exhibit more favorable thermodynamic profiles than conventional hydroalcoholic solvents. The observed reduction in enthalpy and Gibbs free energy suggests more efficient interactions between the solvent and bioactive compounds, resulting in lower energy requirements and greater spontaneity of the extraction process.^23,24^ This behavior can be attributed to the strong hydrogen-bonding network present in NADES, which promotes selective and effective solvation of phenolic compounds.

The addition of EtOH has proven to be a promising strategy, as the solvent is classified as GRAS (Generally Recognized As Safe), ensuring safety for food applications. Additionally, EtOH offers strategic advantages, including low cost, wide availability, and partially renewable origin. When combined with NADES, EtOH reduces the high viscosity characteristic of these solvents, promoting the diffusion of bioactive compounds and enhancing extraction yield. Although NADES/EtOH systems remain underexplored for the extraction of bioactive compounds, studies using deep eutectic solvents (DES), which are analogous to NADES, have provided relevant insights. These studies demonstrated that cosolvents can be added at high proportions (1:1 m/m) without compromising the integrity of the eutectic system, provided that the hydrogen-bonding interactions between the main components are preserved.^25–28^ Furthermore, studies have shown that NADES containing cosolvents, such as water, can achieve extraction yields comparable to or higher than those obtained with 70% EtOH (v/v), a widely used conventional solvent.^29–31^ In addition to phenolic compounds, mixtures of NADES and EtOH have been successfully applied to the extraction of carotenoids from buriti peel,^32^ a typical Amazonian raw material, highlighting the potential of these systems for valorizing regional biodiversity and replacing conventional solvents.

The novelty of this study lies in the development of a hybrid eutectic solvent system in which EtOH acts as a strategic viscosity modulator, rather than merely as a cosolvent. This approach aims to preserve the eutectic network while overcoming the mass transfer limitations typically associated with pure NADES. Given this scenario, this study aims to investigate the extraction of phenolic compounds from the pulp of *M*. *dubia* using three systems: choline chloride:acetic acid, choline chloride:acetic acid + water, and choline chloride:acetic acid + EtOH. The proposed approach seeks to correlate solvent properties with extraction efficiency and evaluate the impact of thermodynamic and operational variables on overall yield. The integrated analysis contributes to the optimization of phenolic compound extraction from *M*. *dubia* and establishes sustainable and reproducible protocols capable of guiding future industrial applications in the production of nutraceuticals. Acetic acid is an organic acid that can be obtained by fermentation; therefore, a mixture of choline chloride and acetic acid can be classified as a NADES.^39^

## 2 Results and Discussion

### 2.1 Solvent Characterization

#### 2.1.1 Rheological Behavior

Figure 1 shows the variation in dynamic viscosity of ChCl:AcOH, ChCl:AcOH + H_2_O, and ChCl:AcOH + EtOH at different temperatures. It is observed that, for all systems, the viscosity decreased with increasing temperature, which is expected due to the reduction in intrinsic viscosity with increasing thermal energy and the consequent weakening of intermolecular interactions.^33^

**Figure 1:**
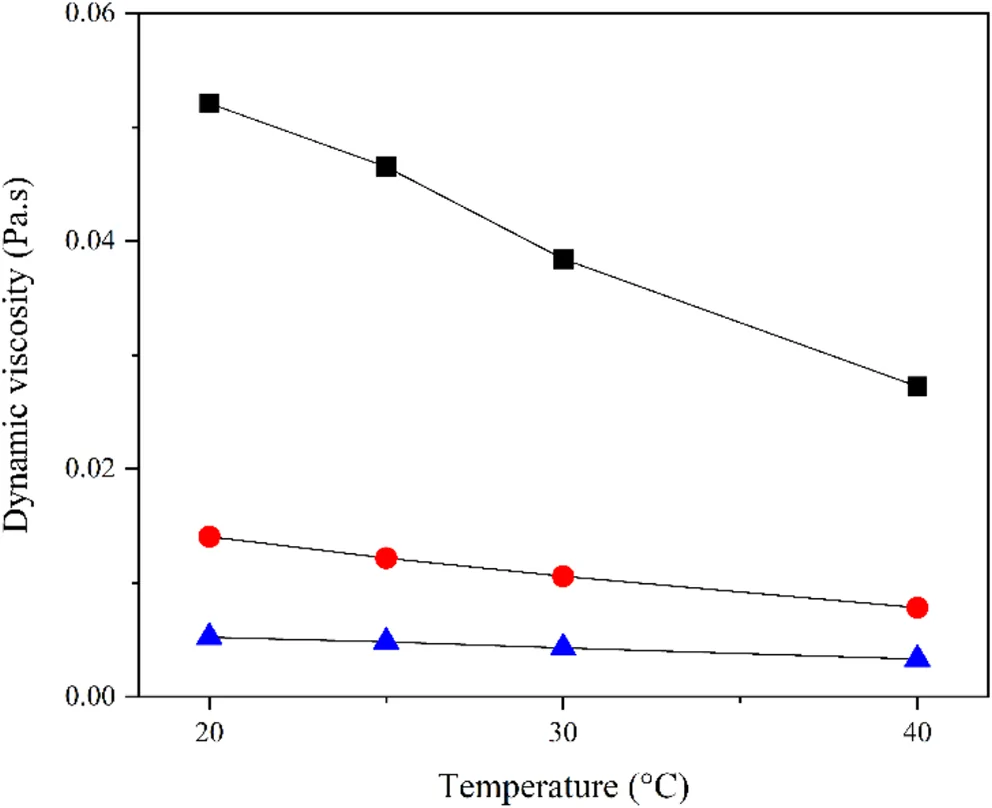
Effect of temperature on the dynamic viscosity of the solvents. (■) ChCl:AcOH, (●) ChCl:AcOH + H_2_O, and (▲) ChCl:AcOH + EtOH.

The experimental values of dynamic viscosity at 40 °C are listed in Table 1. The ChCl:AcOH exhibited the highest viscosity (0.02725 Pa.s), approximately 34 times higher than that of 70% EtOH (0.00080 Pa.s). This difference indicates that, under the same conditions, ChCl:AcOH imposes greater resistance to flow, potentially reducing diffusion rates and hindering solvent mixing and penetration processes in solid matrices. This behavior justifies the need to use water and/or EtOH in the composition of eutectic mixtures to enable extraction applications, where mass transfer is a critical factor. Conversely, the addition of EtOH to ChCl:AcOH reduced the viscosity by approximately 88%, resulting in a system only four times more viscous than EtOH. This reduction favors mass transport and may improve extraction efficiency. Similarly, the ChCl:AcOH + H_2_O mixture showed a 71% decrease in viscosity compared to ChCl:AcOH, indicating a comparable weakening of intermolecular interactions. This result is consistent with previous studies^34^ that demonstrate that hybrid eutectic systems containing up to 50% EtOH can maintain high extraction efficiency and selectivity while exhibiting reduced viscosity, consequently improving mass transfer during the extraction process.

**Table 1:** Dynamic Viscosity of the Solvents at 40 °C^a^

solvent	η (Pa.s)
EtOH^35^	0.00080
ChCl:AcOH	0.02725 ± 0.00061
ChCl:AcOH + H_2_O	0.00783 ± 0.00004
ChCl:AcOH + EtOH	0.00331 ± 0.00004

a EtOH:ethanol; ChCl: choline chloride; AcOH: acetic acid; H_2_O: water.

These values are consistent with the literature,^36^ where the same solvent formulation showed a viscosity value of 0.03 Pa.s at 40 °C. Considering that solvents with viscosity below 0.2 Pa.s favor diffusion and, consequently, the efficiency of the extraction process,^37^ it is observed that both the addition of H_2_O and EtOH make ChCl:AcOH more suitable for application as an extraction solvent.

Eutectic solvents that contain organic acids in their composition tend to have high viscosities, depending on the number of carboxyl groups in their composition. Eutectic mixtures formed by monoprotic acids, such as acetic acid, have lower viscosity when compared to di- and triprotic acids, due to fewer molecular interactions in the medium.^38^ In the literature, NADES formed with ChCl, lactic acid (monoprotic), and citric acid (triprotic) exhibited viscosities of 0.101 and 1.671 Pa.s, respectively.^39^

Table 2 presents the parameters obtained by fitting to the Newtonian model. For ChCl:AcOH and ChCl:AcOH + water, the model showed good fit at all temperatures (*R* ^2^ ≥ 0.998; χ^2^ ≤ 0.003), confirming the typical behavior of Newtonian fluids. For ChCl:AcOH + EtOH, the values of *R* ^2^ were slightly lower (≥0.968) and χ^2^ slightly higher, the Newton model adequately described the data within experimental precision, without showing systematic dependence of viscosity on shear rate in the studied range.

**Table 2:** Parameters and Fitting Results of the Newton Model to ChCl:AcOH, ChCl:AcOH + H_2_O and ChCl:AcOH + EtOH^a^

		Newton’s law		
solvent	temperature (°C)	η (Pa.s)	*R* ^2^	χ^2^
ChCl:AcOH	20	0.05209 ± 0.00015	0.99987	0.00309
	25	0.04652 ± 0.00076	0.99988	0.00221
	30	0.03843 ± 0.00069	0.99994	0.00072
	40	0.02725 ± 0.00061	0.99997	0.00019
ChCl:AcOH + H_2_O	20	0.01405 ± 0.00004	0.99975	0.00057
	25	0.01227 ± 0.00073	0.99881	0.00214
	30	0.01057 ± 0.00006	0.99970	0.00038
	40	0.00783 ± 0.00004	0.99868	0.00081
ChCl:AcOH + EtOH	20	0.00523 ± 0.00001	0.98582	0.00459
	25	0.00482 ± 0.00004	0.98547	0.00352
	30	0.00429 ± 0.00001	0.98127	0.00373
	40	0.00331 ± 0.00004	0.96781	0.00366

a EtOH: ethanol; ChCl: choline chloride; AcOH: acetic acid; H_2_O = water.

Figure 2 presents the result of fitting the Newtonian model to the experimental data for the ChCl:AcOH + EtOH system at 40 °C, indicating that the obtained behavior is consistent with Newtonian flow under the evaluated conditions. The rheological behaviors of the solvents are shown in Figures S1–S3 of the Supporting Information.

**Figure 2:**
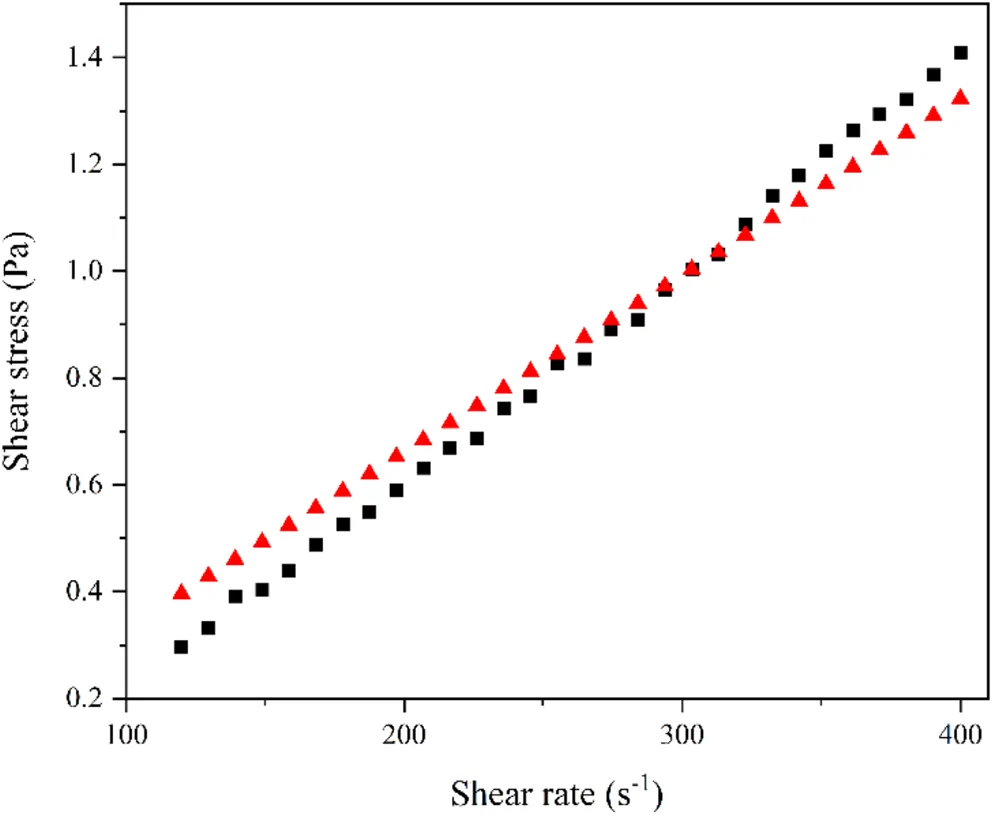
Relationship between shear stress and shear rate of ChCl:AcOH + EtOH at 40 °C. (■) experimental data and (▲) Newton model adjust.

#### 2.1.2 Vibrational Characterization

##### 2.1.2.1 FTIR

Through vibrational characterization by FTIR, it is possible to identify characteristic functional groups of the pure components used in the synthesis of the solvents, as well as observe the bands associated with hydrogen bonds. Figure 3 shows the spectra of the pure components and the studied solvents. The formation of hydrogen bonds is essential for a solvent to be characterized as a NADES and can be evidenced by the shift of typical O–H bands to lower wavenumber regions, along with changes in bandwidth, shape, and intensity.^40^ The redshift observed in the O–H stretching bands is one of the main spectroscopic indicators of hydrogen bond formation in eutectic systems. From an electronic point of view, this phenomenon results from the transfer of electron density from the lone pair orbitals of the hydrogen bond acceptor to the antibonding σ* orbital of the D–H bond (where D represents the donor group).^41^ This interaction increases the population of the antibonding orbital, leading to the stretching and weakening of the D–H bond. Consequently, the vibrational frequency decreases, producing a shift toward lower wavenumbers and providing clear spectroscopic evidence of the establishment of the supramolecular network within the eutectic system.^41^

**Figure 3:**
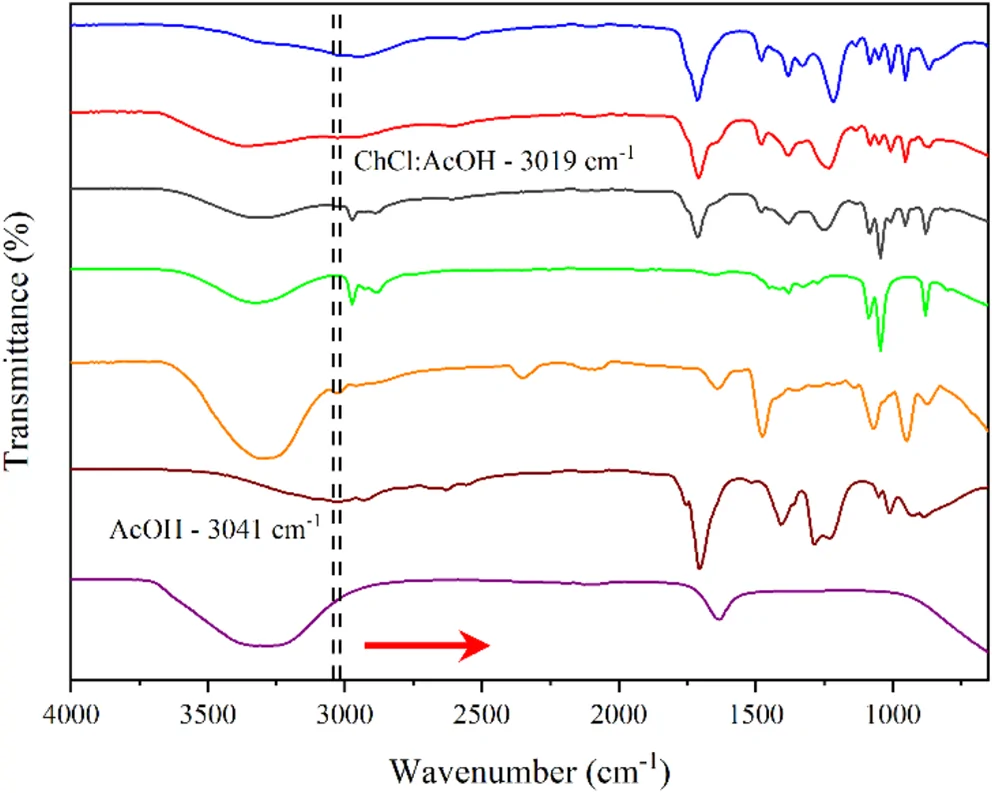
FTIR spectra of the solvents and pure components. (—) ChCl:AcOH, (—) ChCl:AcOH + H_2_O, (—) ChCl:AcOH + EtOH, (—) EtOH, (—) ChCl, (—) AcOH, and (—) H_2_O.

The main bands of ChCl were found at 3310 and 1073 cm^–1^, which refer to the O–H and C–O or C–N vibrational stretches, respectively.^42^ For AcOH, the main characterized bands were 3034, 2930, 1707, 1409, and 1290 cm^–1^, which refer, respectively, to the O–H, C–H, C=O, in-plane COOH deformation (coupling of O–H bending and C–O stretching), and C–O stretches.^43,44^

Water has two bands: an intense one at 3295 cm^–1^ formed by an O–H stretch, and one at 1633 cm^–1^, which is attributed to an H–O–H scissoring deformation.^45^ As for EtOH, it exhibited four identifiable bands: one at 3325 cm^–1^, attributed to O–H stretching vibration; another at 2974 cm^–1^, caused by a C–H stretch; and two at 1044 and 880 cm^–1^, originating from C–O stretching and a C–H deformation, respectively.^46^

The comparative analysis of the spectra confirms the successful formation of NADES (ChCl:AcOH*)*, evidenced by significant modifications in the bands of the individual components. The solvents have characteristics of hydrogen bond formation between their components. The pure NADES showed a redshift of the O–H stretching bands to a lower wavenumber, along with a slight broadening of this band, indicating the establishment of a hydrogen bond network between ChCl and AcOH.

The addition of water to NADES resulted in a reduction in the intensity of the O–H stretching band and a change in its shape, while still retaining traces of the hydrogen bond network. This suggests that water, by interacting with the components of NADES, acts as a competitor for hydrogen bonds, partially disrupting the supramolecular structure of the system.^47,48^ The addition of EtOH caused more pronounced changes: the O–H band in the ChCl:AcOH + EtOH mixture assumes a shape similar to that observed in pure EtOH, indicating disturbance of the original network.^49^ This molecular reorganization explains the drastic reduction in viscosity observed experimentally and suggests a changes in the solvent’s solvation properties, which has direct implications for its performance as an extracting agent.

Although the addition of EtOH promotes molecular reorganization, the persistence of the characteristic C–N (from ChCl) and C=O (from AcOH) bands in the ChCl:AcOH + EtOH spectra confirms that the eutectic structure is preserved. As supported by studies with analogous hybrid solvents,^34^ the hydrogen bonding interactions between HBA and HBD remain stable even in the presence of high cosolvent concentrations, provided that EtOH acts as a lattice modulator, and not as a complete disruptor.

##### 2.1.2.2 Raman Spectroscopy

Raman spectroscopy, like FTIR, revealed detailed information about the intermolecular interactions present in the different solvents, enabling a more complete characterization of the supramolecular structure of the systems. Figure 4 shows the spectrograms obtained from the solvents using Raman spectroscopy.

**Figure 4:**
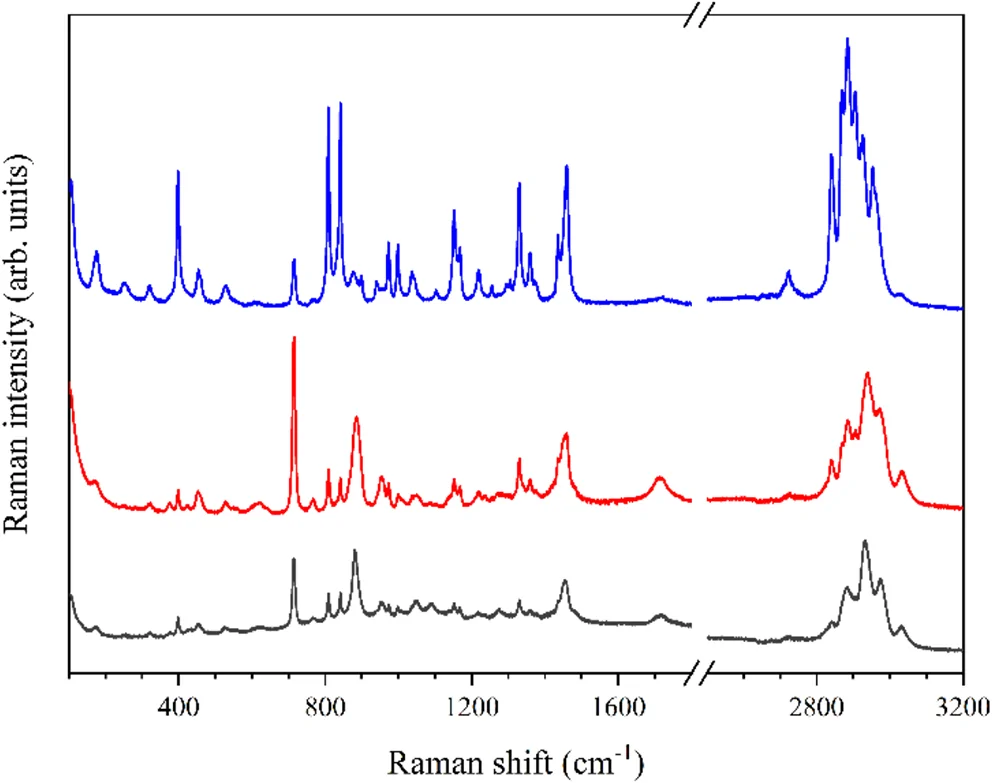
Raman spectra of NADES obtained by Raman spectroscopy. (—) ChCl:AcOH, (—) ChCl:AcOH + H_2_O, and (—) ChCl:AcOH + EtOH.

In the analysis of the region between 400 and 900 cm^–1^, several characteristic bands of the pure components in DES are observed. These peaks are attributed to vibrations of the C–N and C–O–H bonds associated with ChCl and the C–C and C–O–H bonds of AcOH, reflecting the intermolecular interactions established between the individual components of the eutectic mixture.^50,51^ In the binary mixtures with water and EtOH, these peaks are still present, indicating the presence of the same interactions, albeit with reduced intensities that suggest modifications in the local chemical environment.

A band centered around 1460 cm^–1^ was observed for all studied solvents and is attributed to the asymmetric H–C–H stretching vibration of acetic acid.^50^ Because this band is sensitive to the local intermolecular environment, it can be used to monitor changes in the molecular aggregation state of the system.

The analysis of the region from 2500 to 3200 cm^–1^ reveals information about the vibrations associated with C–H and O–H bonds, which are particularly sensitive to hydrogen bonds. In pure DES, the most intense bands are observed in the range of 2800–3000 cm^–1^, attributed mainly to the C–H stretching vibrations of the methyl and methylene groups present in ChCl.^52^ The absence of a significant band above 3000 cm^–1^ suggests that AcOH is involved in hydrogen interactions that suppress its O–H vibrations,^50^ highlighting the strong hydrogen bond network typical of ChCl:AcOH.

The addition of water and EtOH to ChCl:AcOH caused distinct changes in the hydrogen bonding network, complementing the observations made by FTIR. Water caused slight reductions in the intensity of the bands between 400–900 cm^–1^ and at 1460 cm^–1^, along with broadening of the bands 2800–3000 cm^–1^ and the emergence of a band near 3100 cm^–1^, indicating supramolecular disorganization and the formation of new bonds with water. This behavior is consistent with the role of water as a viscosity modulator, competing for hydrogen bonding sites in the supramolecular network.

The addition of EtOH promoted more pronounced spectral changes, including a reduction in the intensity of characteristic ChCl:AcOH bands and the appearance of bands typical of EtOH in the 2950–3000 cm^–1^ region. These modifications provide additional evidence of molecular reorganization within the system and are consistent with the substantial reduction in viscosity observed experimentally.

Together, the Raman and FTIR results provide complementary spectroscopic evidence that explains the physicochemical behavior of the studied solvents. The observed spectral changes establish a clear relationship between the organization of the hydrogen-bond network, the viscosity of the systems, and their performance as extraction media.

### 2.2 Particle Size Characterization of the Raw Material

A crucial factor in the extraction of bioactive compounds is the particle size of the raw material, which directly influences the specific surface area available for contact between the solid and the solvent. Generally, reducing particle size decreases the intraparticle diffusion path and increases the solid–liquid contact area, resulting in higher mass transfer rates and, consequently, greater efficiency in recovering the compounds of interest. The values obtained for the particle size characterization of the freeze-dried and ground pulp of *M*. *dubia* are presented in Table 3.

**Table 3:** Particle Size Distribution and Mean Diameter of Freeze-Dried and Ground *M*. *dubia* Pulp

sieve size (#Tyler)	opening diameter, *D* (mm)	retained mass (g)	mass fraction (*X*_*i*_)
20	0.880	2.59	0.0259
–20 + 28	0.740	5.61	0.0561
–28 + 35	0.513	3.63	0.0363
–35 + 42	0.390	22.61	0.2260
–42 + 48	0.328	39.52	0.3951
–48 + 60	0.275	26.07	0.2606
total		100.03	1.0000
Sauter mean diameter (*D*_32_)	0.344 mm		

Particle size classification revealed a *D*_32_ de 0.344 mm, a parameter widely used as an indicator of the specific surface area available for mass transfer processes. In chemical engineering, the use of *D*_32_ is particularly relevant because the total external area of a collection of particles is inversely proportional to their size, directly influencing the intensity of interactions between the solid matrix and the solvent.^53^ In the case of *M*. *dubia* pulp, this reduced mean diameter favors solvent access to the cellular compartments where bioactive compounds, such as phenolics and other water-soluble antioxidants, are located, contributing to the reduction of mass transfer resistances at the solid–liquid interface.^54^

The particle size distribution also showed that the raw material is predominantly composed of fine fractions, with approximately 88% of the total mass presenting diameters below 0.4 mm and the largest fraction retained on the 0.328 mm aperture sieve (39.51%). This particle size profile is considered favorable for the recovery of antioxidant compounds, as it reflects a high extent of mechanical disruption of the plant matrix promoted during grinding. As a consequence, greater exposure of intracellular structures to the extracting solvent occurs, facilitating the diffusion and solubilization of bioactive compounds.^54^ Furthermore, experimental evidence suggests that part of these compounds may become more accessible to the solvent as early as the grinding stage, favoring the efficiency of the subsequent extraction process.^55^

Although the particle size distribution shows a predominance of fine particles, the presence of different size classes can influence the overall extraction kinetics. Recent studies demonstrate that models based on a single characteristic particle size can result in inaccurate estimates of extraction times, as particles of different dimensions exhibit distinct specific surface areas and diffusional resistances.^55^

In this context, the presence of particles smaller than the mean diameter (26.06% with 0.275 mm) tends to favor the initial stage of extraction through the washing mechanism, which is associated with the rapid removal of compounds located on the surface or in more accessible regions of the plant matrix.^56^ In contrast, the larger particle size fractions (2.59% with 0.880 mm) may present greater internal diffusional limitations, contributing to the reduction of the extraction rate in the final stages of the process.^56^

Therefore, the obtained particle size distribution suggests favorable conditions for mass transfer during extraction, constituting an important parameter for interpreting the experimental results presented in the subsequent sections.

### 2.3 Preliminary Tests

Table 4 shows the values of total phenolic compounds (TPC) in the extracts obtained by ChCl:AcOH, ChCl:AcOH + H_2_O, + ChCl:AcOH EtOH, and 70% EtOH using different extraction methods. UAE was the method that presented the highest extraction yield of these compounds, with 490 W power being the most efficient for ChCl:AcOH + H_2_O and 560 W power being the most efficient for ChCl:AcOH and ChCl:AcOH + EtOH.

**Table 4:** TPC Concentration in *M*. *dubia* Pulp under Different Extraction Processes

	TPC (mg GAE/g)^a^			
extraction method	ChCl:AcOH	ChCl:AcOH + H_2_O	ChCl:AcOH + EtOH	EtOH
CE	–	–	–	14.86 ± 0.07
SE	15.72 ± 0.03^BCb^	15.66 ± 0.06^BCb^	16.07 ± 0.05^Ba^	–
UAE – 420 W	15.61 ± 0.04^Cb^	15.88 ± 0.07^ABab^	16.14 ± 0.09^Ba^	–
UAE – 490 W	15.88 ± 0.04^ABb^	16.03 ± 0.06^Ab^	16.26 ± 0.02^Ba^	–
UAE – 560 W	16.07 ± 0.05^Ab^	15.47 ± 0.04^*Cc*^	17.11 ± 0.09^Aa^	–

a Treatments with different letters in the same row or column differ significantly from each other (*p* < 0.05), according to Tukey’s test.

The solvent that showed the highest extraction efficiency for phenolics was ChCl:AcOH + EtOH, (17.11 mg GAE/g) at a power of 560 W, attributed to the low viscosity of the solvent. Viscosity is a determining factor in the efficiency of bioactive molecule extraction, as high viscosities reduce the solubilization of target compounds due to decreased mass transfer from the sample to the solvent, resulting in low extraction capacity.^37^ Additionally, the supramolecular structure of ChCl:AcOH, characterized by FTIR and Raman analyses, appears to favor specific interactions with phenolic compounds, enhancing the extraction mechanism.

It is worth noting that, although 70% EtOH exhibits a substantially lower viscosity than the ChCl:AcOH + EtOH system, its extraction efficiency was lower. This behavior suggests that extraction is not governed solely by solvent fluidity, but also by the intermolecular interactions established between the solvent and the phenolic compounds. The hydrogen bonding network characteristic of eutectic systems may favor the solubilization and stabilization of these compounds, resulting in higher extraction efficiency than conventional hydroalcoholic solvents. These findings indicate that the ChCl:AcOH + EtOH system combines favorable mass transfer characteristics with specific solvent–solute interactions, contributing to its superior extraction performance.

The percentage comparison between the methods reinforces the superiority of UAE. The use of ChCl:AcOH + EtOH with a power of 560 W resulted in an approximately 15% increase in TPC yield compared to 70% EtOH applied in CE. Compared to SE, the same condition showed a 6.5% increase. Similarly, it was observed that increasing the power from 420 to 560 W promoted a 3% increase in yield compared to ChCl:AcOH. These results reinforce that both the operating conditions and the solvent composition directly impact the efficiency of TPC extraction.

In addition to its superior performance compared to 70% EtOH, the ChCl:AcOH + EtOH system presents characteristics that may be advantageous when compared to other green extraction technologies reported in the literature. Supercritical CO_2_ extraction generally requires high-pressure equipment and a substantial capital investment, while ionic liquid-based systems may face challenges related to solvent synthesis, recovery, and potential toxicity. In contrast, NADES are typically prepared through simple procedures, exhibit low volatility, and are frequently composed of biodegradable constituents recognized as safe for industrial applications.

Although ionic liquids often exhibit high extraction efficiency, their large-scale application remains under discussion due to concerns regarding environmental persistence, solvent recovery, and potential toxicity. For example, the extraction of phenolic compounds from *Arctium lappa* leaves using 1-alkyl-3-methylimidazolium-based ionic liquids resulted in an extraction yield approximately 15% higher than that obtained with conventional solvents.^57^ However, toxicological concerns associated with some imidazolium-based ionic liquids have been reported in the literature,^58^ which may limit their wider industrial implementation.

The TPC data found in the pulp of *M*. *dubia* in this study were higher than those reported in the literature (4.95 mg GAE/g)^59^ and for species of the Myrtaceae family, such as *Eugenia reinwardtiana* (9.25 mg GAE/g) and *Syzygium cumini* (9.95 mg GAE/g).^60^ On the other hand, higher values of phenolic compounds (67.10 mg GAE/g) for *M*. *dubia* were reported in another study.^61^ These variations can be attributed to differences in analytical methods, solvents used, fruit ripeness stage, and the inherent phenolic variability of the Myrtaceae family and the species *M*. *dubia*. In this context, it is important to highlight that *M*. *dubia* fruits are exceptionally rich in ascorbic acid, which can react with the Folin-Ciocalteu reagent and contribute to the recorded values of total phenolic compounds.^62^ However, since all samples were analyzed under the same experimental conditions and using the same analytical method, the relative comparison between the different extraction systems remains valid for evaluating extraction efficiency. Therefore, the superiority observed for the ChCl:AcOH systems remains a consistent indicator of their extraction potential.

The results indicate that the ChCl:AcOH + EtOH system exhibited a higher extraction capacity for TPC compared to the conventional extraction using 70% EtOH. This behavior was also observed in another study, in which the extract of *Zingiber officinale* obtained with a eutectic mixture of betalain and lactic acid showed 20.1 mg GAE/g of phenolics, while the extract obtained with 70% EtOH showed 12.3 mg GAE/g.^63^

### 2.4 Sustainability Analysis

The results obtained in the EcoScale and the Analytical GREEnness Metric (AGREE) for the different solvents and extraction methods studied are presented in Table 5. To calculate the extraction yield (eq 1) of phenolic compounds, the value obtained for 70% EtOH relative to ChCl:AcOH + EtOH was used, as the latter showed higher extraction efficiency and was considered the performance reference. The extractions that exhibited the highest sustainability performance were those conducted by UAE at 560 W. These conditions achieved EcoScale values of 91.00, 91.21 and 92.97, and AGREE scores of 0.62 for the ChCl:AcOH + EtOH, NADES + H_2_O and ChCl:AcOH systems, respectively. In contrast, extraction with 70% EtOH resulted in EcoScale and AGREE scores of 83.43 and 0.52, respectively. Detailed AGREE scores for each of the 12 principles are presented in Table S1 of the Supporting Information.

**Table 5:**
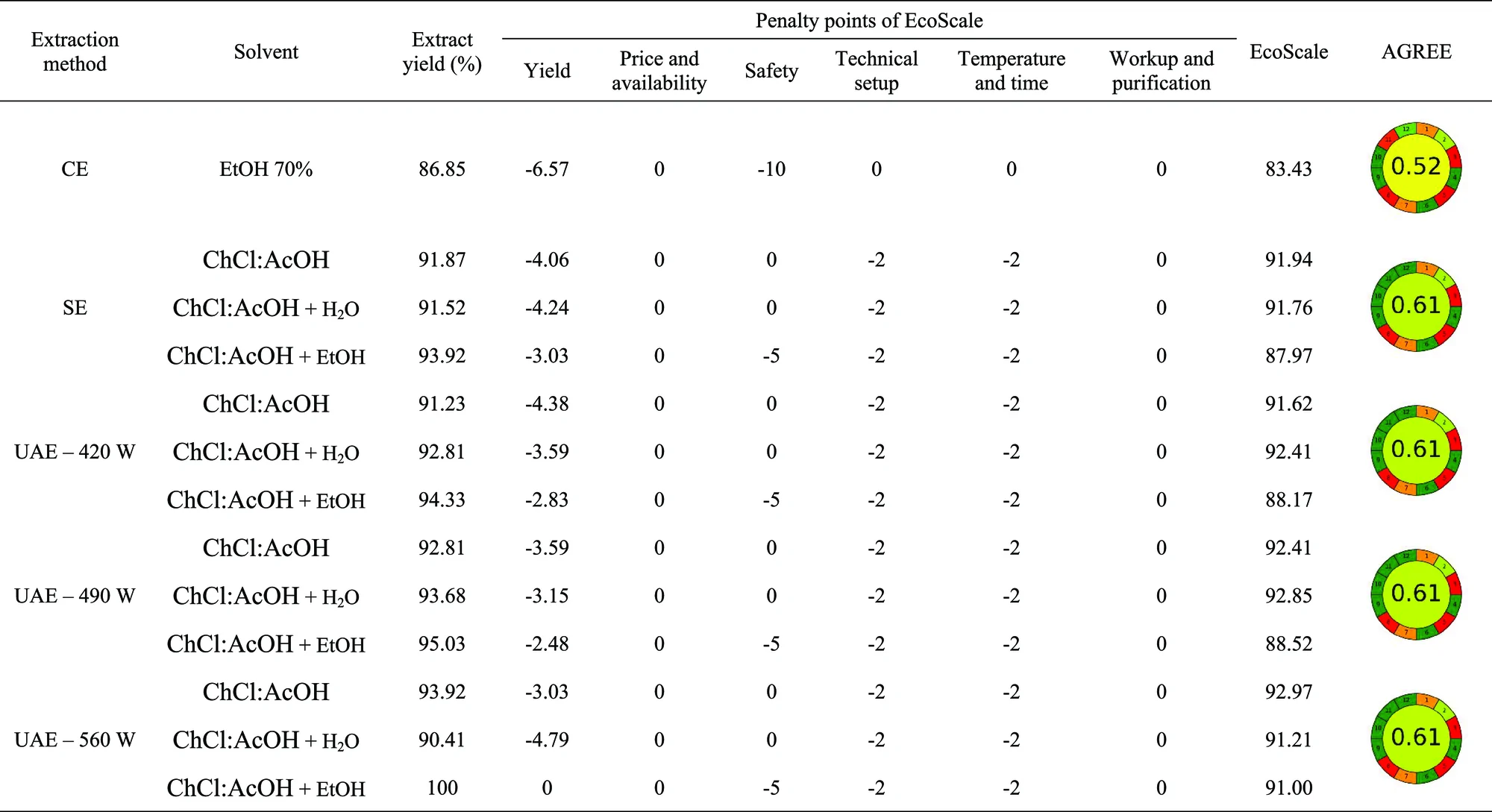
Penalty Points from the EcoScale Assessment and AGREE Results for the Different Extraction Methods and Solvents Employed

1 $$Extraction\;yield\left(\%\right)=\left(\frac{{TPC}_{EtOH70\%{ChCl:AcOH/}_{ChCl:AcOH+water}}}{{TPC}_{ChCl:ACOH+EtOH}}\right)$$

The score obtained for conventional extraction is primarily associated with the safety of the solvent used, EtOH, although it is a renewable-derived solvent and widely employed in the pharmaceutical and food industries, it presents a flammability risk when used in high concentrations.^64^ Additionally, it requires additional purification steps to remove residual solvent, which increases the cost and reduces the sustainability of the extraction process.

The ChCl:AcOH showed the highest safety score on the EcoScale due to its characteristics of low volatility, low flammability, composition from natural and biodegradable compounds, reinforcing its sustainability features. Despite the limitations imposed on EtOH (flammable and potential toxicity), its incorporation into eutectic systems is viewed positively as it contributes to reducing volatility and raising the flash point of the mixture, mitigating the risks associated with the pure conventional solvent. Furthermore, the formation of hydrogen bonding networks in ChCl:AcOH contributes to reducing the vapor pressure of ethanol. However, the ChCl:AcOH + EtOH system was still penalized by 5 points on the safety scale due to the residual flammability of the mixture, considering the molar ratio of EtOH used (1:2:3.8).

An additional advantage of the ChCl:AcOH + EtOH system is the potential for direct incorporation of the resulting extracts into food, nutraceutical, or cosmetic formulations. Because the solvent components are GRAS, the need for extensive solvent removal and purification steps can be reduced, potentially decreasing energy consumption and processing costs compared to conventional extraction systems.

Although the mixture receives a five-point penalty on the EcoScale metric due to residual flammability, this limitation must be considered in conjunction with the potential reduction in downstream processing requirements, which are commonly required when toxic or food-incompatible solvents are employed.

However, it is important to highlight that the direct incorporation of these extracts into food matrices still requires further studies to evaluate the long-term stability of the bioactive compounds, the sensory impacts, and the technological compatibility with the formulations of the final products.

The results found for the AGREE and EcoScale scales in this study are close to those reported in the literature.^65,66^ These results reinforce that the use of ChCl:AcOH combined with cosolvents and modern extraction techniques not only preserves extraction efficiency but also enhances the environmental and operational performance of the process. These findings suggest that the combination of ChCl:AcOH-based systems and modern extraction techniques can improve both extraction performance and sustainability indicators. The results support the potential application of these systems as greener alternatives for the recovery of phenolic compounds.

Although pure ChCl:AcOH exhibited the highest sustainability scores, the ChCl:AcOH + EtOH system coupled with UAE at 560 W was selected for subsequent kinetic and thermodynamic studies because it provided the best balance between extraction efficiency and environmental performance.

### 2.5 Preliminary Economic Analysis

Table 6 presents the estimated capital expenditures (CapEx) for the different extraction processes evaluated. Among the technologies analyzed, UAE presented the lowest total investment cost, while SE required the highest capital investment. The higher cost observed for SE was mainly associated with the acquisition of the shear extraction system, while the lower cost of UAE was attributed to the reduced investment required for the ultrasound equipment.

**Table 6:** Estimated CapEx for the Different Extraction Processes Evaluated

	cost (US$)		
equipment	CE	SE	UAE
freeze-dryer	15,420.08	15,420.08	15,420.08
mill	991.26	991.26	991.26
mixer	1028.01	1550.46	1550.46
agitation system	2218.44		
shear system		14,186.47	
ultrasonic system			7093.24
centrifuge	4531.89	4531.89	4531.89
evaporator and concentrator	10,280.11		
storage tank	1698.13	1308.21	1308.21
installations and plumbing	3616.79	3798.84	3089.51
electrical system and instrumentation	3616.79	3798.84	3089.51
import and delivery	2531.75	2659.19	2162.66
total cost	29,521.92	31,833.89	22,825.48

Regardless of the extraction technology, freeze-drying accounted for the largest contribution to total capital expenditure, representing 48.4 to 67.5% of the overall investment. This result highlights the significant economic impact of pilot-scale raw material stabilization and dehydration operations.

The conventional extraction process required an additional evaporation and concentration unit for solvent removal, which increased capital expenditure. In contrast, ChCl:AcOH-based systems were designed to produce ready-to-use extracts, eliminating need for solvent recovery and concentration steps, thus simplifying process setup and reducing equipment requirements.

Operational expenditure (OpEx) and cost of manufacturing (COM) are presented in Table 7. The cost of raw material (CRM) represented the largest share of operating expenses, corresponding to approximately 84.7% of total OpEx (sum of CRM, CUT, COL, and CWT). On the other hand, cost of utilities (CUT) and cost of operating labor (COL) showed relatively small contributions, indicating that the overall process economy is strongly dependent on the cost of the inputs used. COM reflected the combined impact of these operating expenses associated with the depreciation rates of each technology’s CapEx.

**Table 7:** Economic Indicators for Pilot-Scale Production of Ready-to-Use Extracts from *M*. *dubia*

		OpEx (US$/year)						
system	solvent	CRM	CUT	COL	CWT	COM (US$/year)	extract production (kg/year)	extract cost (US$/kg)
CE	EtOH 70%	84,896.46	4689.68	10,611.26	0.00	120,842.79	66,000	1.83
SE	ChCl:AcOH	84,896.46	4735.76	10,611.26	0.00	148,893.89	66,000	2.26
	ChCl:AcOH + H_2_O	84,896.46	4735.76	10,611.26	0.00	137,651.53	66,000	2.09
	ChCl:AcOH + EtOH	84,896.46	4735.76	10,611.26	0.00	143,510.85	66,000	2.17
UAE	ChCl:AcOH	84,896.46	4689.68	10,611.26	0.00	146,098.65	66,000	2.21
	ChCl:AcOH + H_2_O	84,896.46	4689.68	10,611.26	0.00	134,856.29	66,000	2.04
	ChCl:AcOH + EtOH	84,896.46	4689.68	10,611.26	0.00	140,715.61	66,000	2.13

Among the systems evaluated, the addition of water to ChCl:AcOH promoted a consistent reduction in COM. For processes conducted by SE, the use of ChCl:AcOH containing water reduced COM from US$ 148,893.89 to US$ 137,651.53 per year, while for UAE a reduction from US$ 146,098.65 to US$ 134,856.29 per year was observed. This behavior is related to the lower utilization of ChCl:AcOH components and the reduced cost of water.

The unit cost of producing the extracts ranged from US$1.83 to US$2.26/kg. Although the extract obtained with EtOH had the lowest production cost, followed by the ChCl:AcOH + H_2_O systems using EAU and SE, economic viability should be analyzed in conjunction with product quality.

Figure 5 highlights this relationship between production cost and the total phenolic content obtained. The conventional process occupied the lowest cost region, but exhibited the lowest phenolic content. In contrast, all ChCl:AcOH-based systems achieved higher concentrations of bioactive compounds, justifying the higher operational cost compared to the added value of the extract obtained. Among the treatments evaluated, the ultrasound-assisted ChCl:AcOH + EtOH system presented the best balance between production cost and extraction efficiency, providing a favorable equilibrium between technical performance and economic viability.

**Figure 5:**
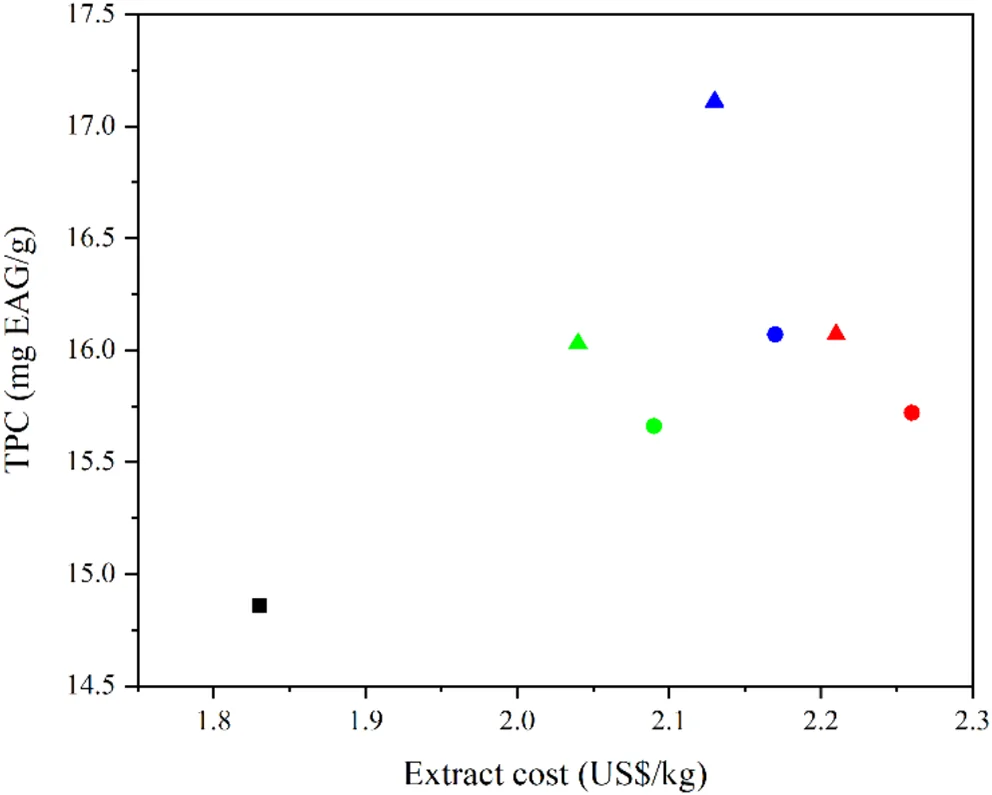
Relationship between extract production cost (US$/kg) and total phenolic content (TPC) of extracts obtained using different extraction systems. (■) EtOH 70%, (●) ChCl:AcOH + H_2_O – SE, (▲) ChCl:AcOH + H_2_O – UAE, (●) ChCl:AcOH + EtOH – SE, (▲) ChCl:AcOH + EtOH – UAE, (●) ChCl:AcOH – SE, and (▲) ChCl:AcOH – UAE.

### 2.6 Extraction Kinetics of TPC

The extraction kinetics of TPC from the pulp of *M*. *dubia* at different temperatures, using ultrasound and ChCl:AcOH + EtOH, are presented in Figure 6. The initial stage of the extraction process is dominated by a washing mechanism, in which the solvent readily solubilizes phenolic compounds located on the particle surface. As a result, the extraction yield increases sharply during the early stages of the process until equilibrium is gradually approached. This behavior is characteristic of solid–liquid extraction systems involving plant matrices.

**Figure 6:**
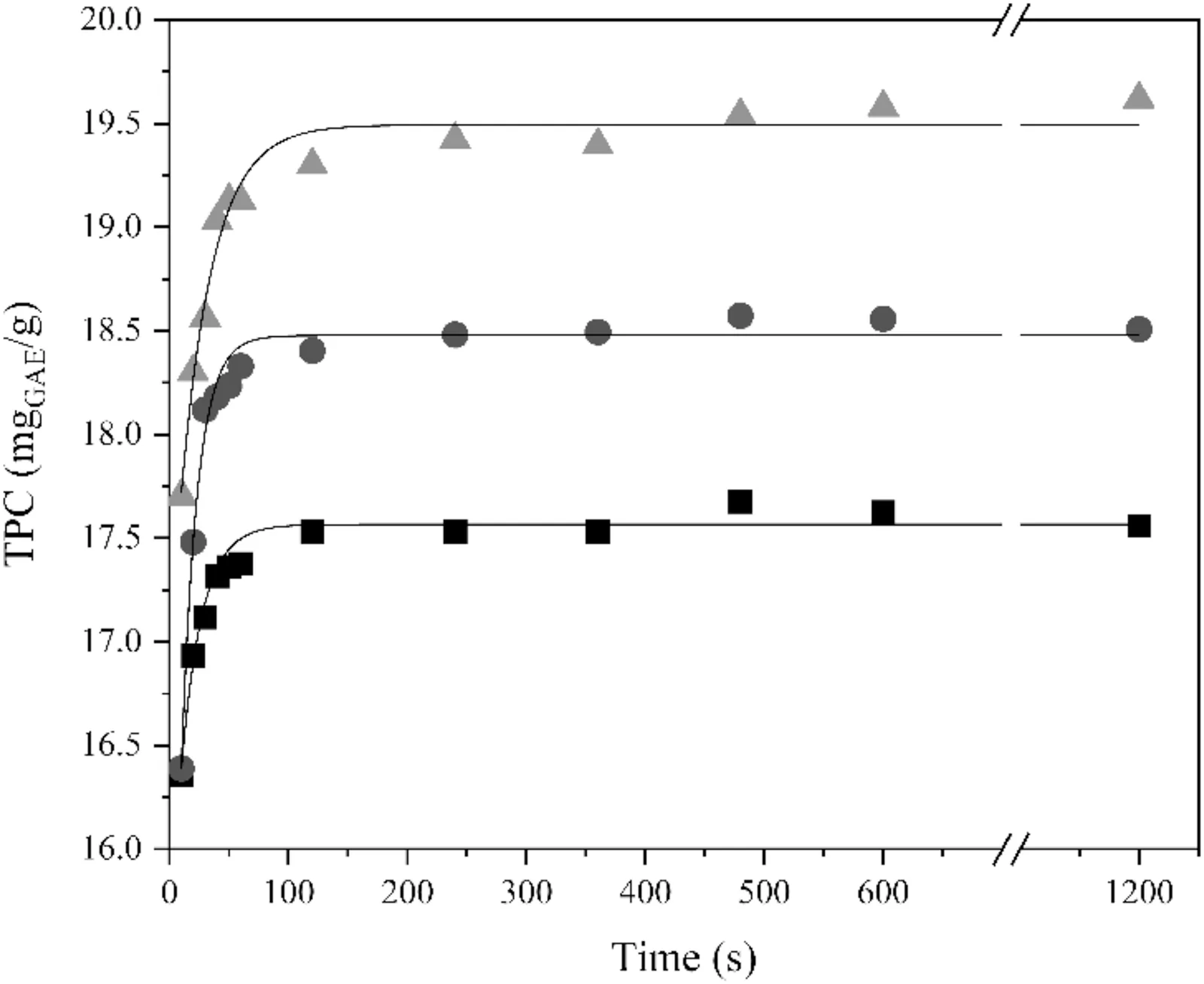
Extraction kinetics of TPC from *M*. *dubia* fruit. (■) 20 °C, (●) 30 °C, and (▲) 40 °C.

An increase in temperature positively affected the extraction process. This behavior can be attributed to the higher solubility of phenolic compounds and the reduction in solvent viscosity at elevated temperatures, both of which facilitate the diffusion of phenolics from the solid matrix into the liquid phase.

Temperature may also influence the efficiency of ultrasound-assisted extraction by enhancing acoustic cavitation phenomena. More intense cavitation can promote greater disruption of plant cell structures, accelerate the initial extraction rate, and contribute to higher equilibrium yields.

The combined effect of improved mass transfer and intensified cavitation provides a plausible explanation for the higher extraction efficiencies observed at elevated temperatures. Similar behavior has been reported in previous studies involving the ultrasound-assisted extraction of bioactive compounds.^67,68^

### 2.7 Mathematical Modeling

The evaluation of the applied mathematical models revealed not only differences in predictive performance but also valuable insights into the physical mechanisms governing the extraction process. Analysis of the model parameters (Tables 8 and 9) as a function of temperature provided a deeper understanding of the influence of thermal conditions on mass transfer and extraction kinetics.

**Table 8:** Mathematical Parameters of the Five Kinetic Models Used for the Extraction of TPC from *M*. *dubia*

models	Fick’s law		PSO^b^		partition				Weibull sorption			Peleg’s sorption		
parameters^a^	*A*	*B*_1_	*h*	*k*_2_	α	*a*	β	*b*	*A*	*k*_w_	*m*	*K*_1_	*K*_2_	*B*_0_
temperature														
20 °C	0.1382	0.0674	18.770	0.0599	14.770	1.248	2.786	0.087	17.601	1.190	0.347	0.055	0.056	18.182
30 °C	0.2498	0.0789	21.734	0.0627	14.792	1.165	3.653	0.071	18.500	1.201	0.296	0.052	0.054	19.231
40 °C	0.2430	0.0940	24.972	0.0663	15.803	0.901	3.702	0.052	19.567	1.285	0.260	0.051	0.051	19.608

a Units: *A* (dimensionless); *B*_1_ (s^–1^); h (mg GAE.s/g); *k*_2_ (g/mg GAE.s); α (mg GAE/g); *a* (s^–1^); β (mg GAE/g); *b* (s^–1^); *A* (mg GAE/g); *k*_w_ (s^–1^); *m* (dimensionless); *K*_1_ (mg GAE.s/g); *K*_2_ (g/mg GAE); and *B*_0_ (mg GAE/g.s);

b Pseudo-second order.

**Table 9:** Statistical Parameters of the Five Kinetic Models Used for the Extraction of TPC from *M*. *dubia*

models	Fick’s law		PSO		partition		Weibull sorption		Peleg’s sorption	
adjustment	*R* ^*2*^	χ^*2*^	*R* ^*2*^	χ^*2*^	*R* ^*2*^	χ^*2*^	*R* ^*2*^	χ^*2*^	*R* ^*2*^	χ^*2*^
temperature										
20 °C	0.9877	0.0031	0,9300	0,0110	0.9697	0.0584	0.9857	0.0036	0.9235	0.0146
30 °C	0.9864	0.0083	0,8669	0,0543	0.9664	0.0161	0.9276	0.0344	0.9175	0.0393
40 °C	0.9523	0.0689	0,8680	0,0450	0.9071	0.0316	0.9764	0.0095	0.9437	0.0189

The Fick’s law model stood out as the most robust (*R* ^*2*^ > 0.9523; χ^*2*^ < 0.069), and the analysis of its parameters corroborates the diffusive nature of the process. The consistent increase in the diffusion rate constant (*B*_1_) with temperature (from 0.0674 s^–1^ at 20 °C to 0.0940 s^–1^ at 40 °C) reflects the increase in the effective diffusion coefficient (*D*_eff_) of phenolic compounds. This behavior is characteristic of diffusion-controlled processes, where increasing temperature reduces solvent viscosity and increases molecular kinetic energy, facilitating solute migration from the matrix to the solution. This behavior is consistent with previous studies, which observed a linear increase in diffusivity with temperature during ultrasonic extraction of antioxidants from *Fagopyrum tataricum*.^69^

In contrast, the PSO model showed the lowest accuracy (*R* ^*2*^ = 0.866 to 0.93; χ^*2*^ = 0.011 to 0.045). The parameter *h*, which increased from 18.77 to 24.972 mg GAE.s/g with temperature, confirms the thermal influence on the initial kinetics. The slight increase in *k*_*2*_ (0.0599 to 0.0663 g/mg GAE.s) suggests a modest improvement in the adsorption/extraction rate during the final stages. Similar results were found in the literature for the extraction of bioactive compounds from *Moringa oleifera* leaves.^6^ However, the empirical and simplified nature of the model proved limiting for describing the complete extraction profile of such a complex system. Although widely applied, the PSO model does not necessarily represent a true chemisorption mechanism and may simply reproduce kinetic trends associated with intraparticle diffusion or other mass-transfer phenomena.^70^

The partition model, despite its complexity (four parameters), maintained good performance (*R* ^*2*^ between 0.9071 and 0.9697). The increase in α (14.77 to 15.803 mg GAE/g) and β (2.786 to 3.702 mg GAE/g) with temperature indicated greater extraction capacity in both compartments of the plant matrix. However, the simultaneous decrease in the constants *a* (1.248 to 0.901 s^–1^) and *b* (0.087 to 0.052 s^–1^) suggests a more complex extraction mechanism. At higher temperatures, the contribution of multiple extraction compartments may become more pronounced, resulting in an apparent redistribution of the kinetic contributions represented by these parameters.

The good performance of the Weibull model (*R* ^*2*^ ≥ 0.9276) is attributed to its flexibility. The shape parameter *k*_w_ close to 1, combined with the increase of the parameter *A* (17.601 to 19.567 mg GAE/g) with temperature, suggests predominantly simple exponential kinetics, but with a slight increase in system heterogeneity at higher temperatures. The versatility of the Weibull model to describe such behaviors was confirmed in previous studies involving ultrasound-assisted extractions of polyphenols.^9^

The reliability of the Peleg model (*R* ^*2*^ = 0.9175 to 0.9437) is evidenced by the consistent trend of its parameters. The decrease in *K*_1_ (0.347 to 0.26 mg GAE.s/g) indicates an increase in the initial extraction rate with temperature, while the small variation in *K*_2_ (0.056 to 0.051 g/mg GAE) suggests stability in the maximum extraction capacity. The increase in *B*_0_ (18.182 to 19.608 mg GAE/g.s) corroborates the higher initial extraction rate observed at elevated temperatures of the process at elevated temperatures, a pattern consistently observed in bioactive extraction studies.^71^

The overall performance of the evaluated models suggests that the extraction process is predominantly controlled by diffusion, with kinetic behavior reflecting the structural heterogeneity of the plant matrix. The superior performance of the Fick model, along with the satisfactory fit of the Weibull, Peleg, and partition models, indicates that mass transfer plays a central role in the extraction mechanism, while additional phenomena associated with matrix complexity, solvent–solute interactions, and the eutectic system may also contribute to the observed kinetic profile.

### 2.8 Mass Transfer Parameters

The kinetic data obtained from the extraction experiments were used to estimate the *D*_eff_, the mass transfer coefficient (*k*_T_), and the *B_i_* based on Fick’s diffusion model (Table 10). These parameters provide valuable information regarding the mechanisms governing mass transfer during the extraction process and the relative contributions of internal and external diffusion resistances.

**Table 10:** Mass Transfer Parameters of TPC Obtained by UAE from *M*. *dubia* under Different Extraction Conditions

Mass transfer parameters			
temperature (°C)	*D*_eff_ (m^2^/s)	*k*_T_ (m/s)	*B*_*i*_
20	1.93 × 10^–10^	1.96 × 10^–4^	170.70
30	2.26 × 10^–10^	2.20 × 10^–4^	164.15
40	2.69 × 10^–10^	2.43 × 10^–4^	151.96

The value of *D*_eff_ increased with increasing temperature during the sonication process, indicating enhanced mass transfer of phenolic compounds from the plant matrix into the solvent and a reduction in the time required to approach extraction equilibrium. This behavior is likely associated with the decrease in solvent viscosity and the increase in molecular mobility at higher temperatures. Similar behavior has been reported for the extraction of biomolecules from Vaccinium bracteatum leaves, where *D*_eff_ values increased from 3.47 × 10^–12^ to 10 × 10^–12^ m^2^/s as the temperature increased from 50 to 70 °C, respectively.^72^

The values of *k*_T_ exhibited behavior similar to that observed for *D*_eff_, confirming the expected relationship between diffusional transport and the overall mass transfer coefficient in liquid media. The increase in *k*_T_ with temperature can be attributed to greater molecular mobility, resulting from the increase in thermal energy and the reduction in the dynamic viscosity of the medium. The values obtained in this study are within the same range reported for the solid–liquid extraction of protopine from *Fumaria officinalis* (4.8 × 10^–6^ m/s to 11.6 × 10^–3^ m/s; 20 to 70 °C) using different concentrations of EtOH.^73^

The *B*_*i*_ expresses the relative importance of internal and external mass transfer. High values of *B*_*i*_ (between 50 and 100) indicate that the internal resistance is predominant and, therefore, the extraction process is mainly controlled by diffusion.^73^ On the other hand, low values of *B*_*i*_ (≤50) indicate the predominance of external resistance.

The *B*_*i*_ values obtained in this study (151.96–170.70) were substantially higher than the limit commonly associated with diffusion-controlled extraction processes, indicating that external resistance was insignificant compared to internal diffusion resistance. These results confirm that the extraction process was predominantly governed by solute diffusion within the plant matrix. A slight decrease in *B_i_* was observed with increasing temperature, from 170.70 at 20 °C to 151.96 at 40 °C. This trend suggests that the contribution of internal resistance became slightly less pronounced at higher temperatures due to increased diffusivity. However, internal diffusion remained the dominant mass transfer mechanism throughout the entire temperature range evaluated.

The predominance of resistance to internal diffusion can be attributed to the intense agitation generated during ultrasound-assisted extraction. Acoustic cavitation promotes microturbulence and continuous solvent renewal on the particle surface, reducing the thickness of the outer boundary layer and minimizing external mass transfer limitations.^74^ Consequently, the overall extraction rate becomes primarily regulated by the diffusion of phenolic compounds within the plant matrix. Similar high *B*_*i*_ values have been reported for ultrasound-assisted extraction of total phenolic compounds from Syzygium cumini pulp, ranging from 639.47 to 528.19 in the temperature range between 40 and 70 °C.^75^

### 2.9 Thermodynamic Parameters and Activation Energy

The thermodynamic parameters of the UAE process for TPC of *M*. *dubia* are presented in Table 11. The high coefficient of determination (*R* ^*2*^ = 0.9213) obtained from the Van’t Hoff equation (Figure 7) confirms the suitability of the model for estimating thermodynamic parameters. The positive values of enthalpy (Δ*H*) and entropy (Δ*S*) indicate the endothermic nature of the process and the increase in system disorder, respectively. This endothermic character can be partially attributed to the energy required to break solute-matrix interactions, as well as the thermal input resulting from the conversion of ultrasonic energy. The increase in entropy is associated with the transition of compounds from a more ordered structure in the solid phase to the less organized liquid phase.^76^ The spontaneity of the process is confirmed by the negative values of Gibbs free energy (Δ*G*), which become more negative with increasing temperature, indicating greater thermodynamic feasibility at elevated temperatures.

**Table 11:** Thermodynamic Parameters of the Extraction Kinetics of *M*. *dubia*

temperature		thermodynamic parameters			
°C	K	*k*_e_	Δ*H* (kJ/mol)	Δ*S* (J/mol K)	Δ*G* (kJ/mol)
20	293.15	1.249	8.496	30.679	–0.4947
30	303.15	1.325			–0.8014
–0	313.15	1.562			–1.1081

**Figure 7:**
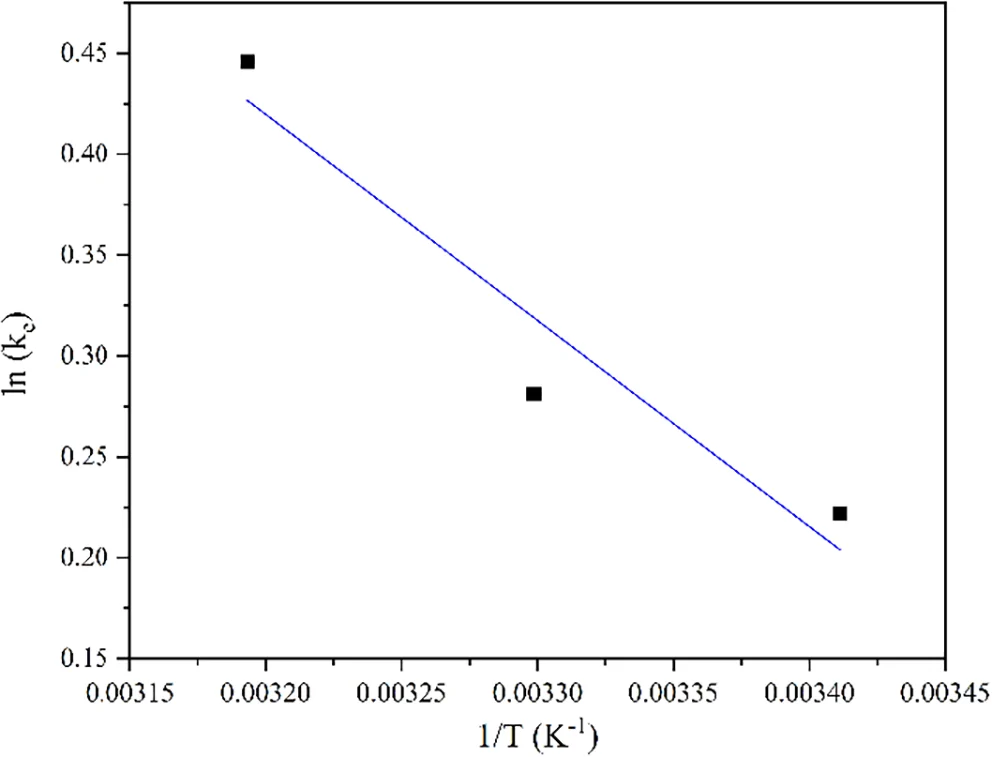
Determination of Δ*H* and Δ*S* as a function of the equilibrium constant (*k*_e_) in the extraction of TPC from *M*. *dubia* at different temperatures.

The values of Δ*H* and *E*_a_ obtained in this study were higher than those reported for other matrices in the literature (Δ*H* = 8.04 kJ/mol; *E*_a_ = 11.747 kJ/mol; Δ*H* = 5.04 kJ/mol).^77,78^ This behavior suggests that the extraction of TPC from *M*. *dubia* using NADES/EtOH requires greater energy input, possibly due to more intense interactions between the solutes and the plant matrix, or the inherent nature of the solvent system employed.

The value of *E*_a_ determined (12.471 kJ/mol), in addition to corroborating the endothermic nature of the process, remains below 20 kJ/mol, indicating that the extraction is predominantly controlled by diffusion.^79,80^ This mechanism is consistent with observations for pomegranate bagasse (*E*_a_ = 14.541 kJ/mol),^81^ reinforcing the applicability of the diffusion model. The high correlation obtained in the fitting of the Arrhenius equation (*R* ^2^ = 0.9978; Figure 8) confirms the suitability of the model to describe the dependence of the extraction rate on temperature.

**Figure 8:**
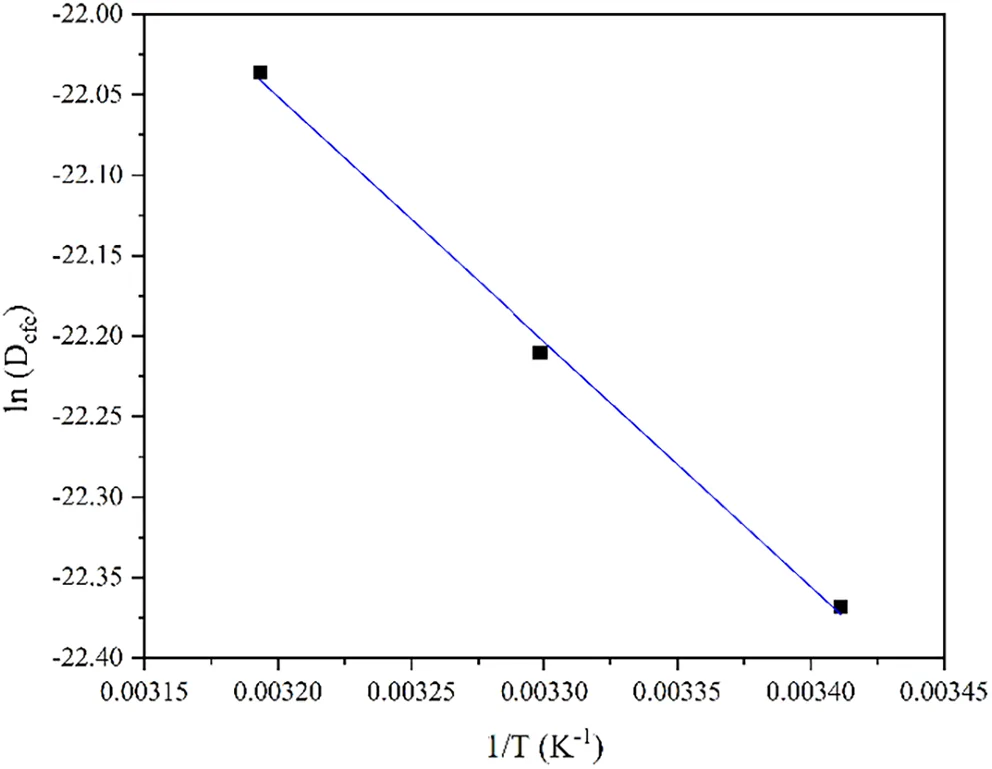
Determination of *E*_a_ in the UAE of TPC from M. dubia at different temperatures using ChCl:AcOH + EtOH.

## 3 Conclusion

The solvents evaluated in this study exhibited suitable rheological and vibrational properties for the extraction of TPC from the *M*. *dubia* pulp. FTIR and Raman analyses provided spectroscopic evidence of the hydrogen-bonding network characteristic of the eutectic system and indicated that the addition of water and EtOH preserved the main structural features of the ChCl:AcOH, while modifying its physicochemical properties.

Among the evaluated conditions, UAE using the ChCl:AcOH + EtOH system resulted in the highest TPC yield. This performance was associated with the combined effects of reduced solvent viscosity and ultrasound-induced cavitation, which enhanced mass transfer during extraction.

Kinetic, mass transfer, and thermodynamic analyses consistently indicated that the extraction process was predominantly controlled by diffusion. Increasing the temperature promoted higher diffusion coefficients, mass transfer coefficients, and extraction yields, while activation energy and Biot number values reinforced the central role of diffusional mechanisms in the extraction process.

The combination of ChCl:AcOH + EtOH proved to be a promising alternative for the extraction of phenolic compounds from camu–camu pulp, exhibiting superior extraction performance and competitive production costs compared with the conventional solvent system. In addition to the higher phenolic content obtained, this mixture provides operational advantages such as reduced viscosity, easier handling, and potential for reuse, contributing to process sustainability.

The preliminary economic assessment indicated that ChCl:AcOH-based systems maintained competitive production costs while enabling the production of ready-to-use extracts. Therefore, the combination of ChCl:AcOH + EtOH and UAE represents a promising strategy for future industrial applications, aligning extraction efficiency, economic performance, and environmental sustainability.

By employing safe and low-cost components, the process enables the production of ready-to-use extracts, in accordance with the principles of green chemistry and the demands of industrial efficiency. Future studies should prioritize the evaluation of these extracts in industrial-scale applications, focusing on the management of residual flammability and the stability of the final products, in order to fully explore the potential of this sustainable extraction strategy.

## 4 Material and Methods

### 4.1 Raw Material

The freeze-dried camu–camu pulp used in this study was kindly donated by 100% Amazônia.

#### 4.1.1 Particle Size Characterization of the Raw Material

The freeze-dried camu–camu pulp was previously ground in a commercial food processor (HR7304/90, Philips Walita, 1000 W). Subsequently, the particle size characterization of the material was performed using a mechanical sieve shaker equipped with a stack of overlapping sieves (Tyler series). The sieves were arranged in decreasing order of mesh opening size, spanning a range from #20 to #60 mesh. Aliquots of 100 g of the sample were placed on the top of the sieve stack and subjected to agitation for 10 min. After the test, the mass retained on each sieve was determined by direct weighing. The mass fractions (*X*_*i*_) corresponding to each particle size interval were calculated based on the total recovered mass. The mean particle size was expressed as the Sauter mean diameter (*D*_32_), determined according to eq 2.^53^

2 $$D_{32}=\frac{1}{\sum_{i=1}^{n}\left(\frac{X_{i}}{D_{\mathrm{pi}}}\right)}$$

### 4.2 Reagents

The reagents used were: ChCl (C_5_H_14_ClNO, ≥98%, ACS Científica, Brazil), acetic acid (AcOH) (C_2_H_4_O_2_, ≥99.7%, Êxodo Científica, Brazil), EtOH (C_2_H_6_O, 96°GL, Prolink, Brazil), anhydrous sodium carbonate (CNa_2_O_3_, ≥99.5%, Synth, Brazil), *Folin*-*Ciocalteu* reagent (Êxodo Científica, Brazil), and ultrapure water (Milli-Q Simplicity UV, Merck Millipore, Germany).

### 4.3 Preparation of ChCl:AcOH

The ChCl:AcOH was prepared according to the methodology proposed in the literature,^82^ using ChCl and AcOH in a 1:2 molar ratio. Predetermined amounts of these components were weighed in a glass beaker, and the mixture was stirred on a magnetic stirrer (SL-51, 300 W, Solab, Brazil) at 70 °C for approximately 60 min, until a viscous and colorless liquid was formed. To prepare the hybrid solvent systems, ultrapure water or EtOH (96% v/v) was added to the prepared NADES according to the proportions established in Table 12. For comparison purposes, a conventional 70% EtOH (v/v) solution was used as a control solvent.

**Table 12:** Proportions of the Reagents Used in the Preparation of the Extraction Solvents

solvent	molar ratio	w/w
ChCl:AcOH	1:2	
ChCl:AcOH:H_2_O	1:2:2.58	
ChCl:AcOH:EtOH	1:2:3.8	
EtOH:H_2_O		70:30

The specific molar ratio of 1:2:3.8 (ChCl:AcOH:EtOH) was defined to create a hybrid solvent system where ethanol acts as a strategic viscosity modulator. This proportion was selected based on literature recommendations for NADES dilution, which demonstrate that adding molecular cosolvents up to a 40–50% (w/w) threshold drastically reduces the high inherent viscosity of the eutectic mixture, thereby enhancing mass transfer kinetics and making the extraction viable.^34,40^ While water is conventionally used for this purpose, 96% EtOH was chosen to modulate the solvent polarity specifically for the extraction of phenolic compounds, optimizing target analyte solubility while preserving the supramolecular hydrogen-bonded network of the ChCl:AcOH.

### 4.4 Characterization of ChCl:AcOH

#### 4.4.1 Rheological Behavior

The rheological behavior of the solvents was characterized using a rheometer (R/S-SST, Brookfield) coupled with an ultrathermostatic bath (RE 206, Lauda, Germany), with a coaxial cylinder measurement system CC-40, at temperatures of 20, 25, 30, and 40 °C. Shear stress and viscosity were obtained by the controlled shear rate (CSR) method, with shear rate ranging from 0 to 400 s^–1^. The rheological analysis was conducted following the methodology described in the literature,^40^ with adaptations in the temperature range and operating conditions employed in the present study.

To obtain the dynamic viscosity, the data obtained from the relationship between shear stress and shear rate were fitted to the Newtonian viscosity law model (eq 3) using nonlinear regression. The statistical parameters used to evaluate the model fit were the coefficient of determination (*R* ^2^) and the chi-square (χ^2^).

3 τ=η×γ̇

Where τ is the shear stress (Pa); η is the dynamic viscosity (Pa.s); γ̇ is the shear rate (s^–1^).

#### 4.4.2 FTIR Spectroscopy

The vibrational characterization of the solvents was performed on an FTIR spectrometer (Cary 630, Agilent Technologies) equipped with a attenuated total reflectance (ATR) ZnSe system, set to 64 scans, using a resolution of 16 cm^–1^ and a spectral range from 4000 to 650 cm^–1^. The analyses were conducted based on a methodology previously described in the literature,^19^ with adaptations to the instrumental conditions used in this study.

#### 4.4.3 Raman Spectroscopy

The solvents were characterized by a Raman spectrometer (LabRAM HR Evolution, Horiba Scientific, Japan) equipped with a charge-coupled device (CCD). The optical system used was a microscope (BX41, Olympus, Japan) equipped with a 20× achromatic objective lens with a numerical aperture (NA) of 0.25, a 633 nm laser, and a power of 5 mW. Data were obtained at a resolution of 2 cm^–1^. The experimental parameters were defined based on methodology reported in the literature,^19^ with adjustments for the systems evaluated in this work.

### 4.5 Extraction Process Selection

#### 4.5.1 Preliminary Tests

To select the most suitable extraction method for the pulp of *M*. *dubia*, three processes were evaluated: conventional extraction (CE), shear extraction (SE), and ultrasound-assisted extraction (UAE). All assays performed in triplicate used a 1:20 ratio (solute:solvent), extraction time of 5 min, and temperature of 40 °C. CE was conducted in a roller shaker (K45-8022, Kasvi, Brazil); SE was performed using a rotor-stator homogenizer (Turratec TE-102, Tecnal, Brazil); and UAE utilized an ultrasonic system (Q700, Qsonica Sonicators) with a 12.7 mm diameter solid titanium probe (reference number: 4219) operating at a fixed frequency of 20 kHz. The ultrasonic power was varied between 420, 490, and 560 W, and the system was coupled to a refrigerated circulator bath (SL-152, Solab, Brazil) for temperature control.

The selection of the extraction process was carried out using the TPC content of the extracts as the response.

#### 4.5.2 Characterization of the Extracts

The TPC of the extracts were quantified using the *Folin*-*Ciocalteu* methodology in a UV/vis spectrophotometer (Evolution 60S, Thermo Scientific) at 765 nm.^83^ For this, 1680 μL of ultrapure water, 20 μL of extract, and 100 μL of 10% *Folin*-*Ciocalteu* reagent were added. After 3 min, 200 μL of 20% sodium carbonate was added, vortexed, and allowed to react for 60 min. The assays were performed in triplicate, with their results expressed in milligrams of gallic acid equivalent per gram of sample (mg GAE/g).

### 4.6 Sustainability Assessment

To assess the sustainability of extraction processes, two tools were employed: EcoScale, which assigns penalty points based on criteria such as yield, cost, safety, technical setup, temperature, time, and purification steps, with the sum subtracted from 100, indicating increasing sustainability from 1 to 100;^84^ and the Analytical GREEnness Metric (AGREE), based on the 12 principles of green analytical chemistry, which expresses the method sustainability through a pictogram with a score between 0 (less green) and 1 (more green).^85^

### 4.7 Preliminary Economic Assessment

A preliminary economic analysis was conducted on a pilot scale to assess the feasibility of producing extracts rich in phenolic compounds obtained from *M*. *dubia* using different extraction systems. The production capacity of the plant was sized for a of 200 kg/day of ready-to-use extract.

Capital expenditure (CapEx) was estimated from quotations obtained from industrial suppliers and specialized commercial platforms. The acquisition costs of the main process equipment were considered, including freeze-dryer, extraction reactor, ultrasound system, centrifuge, solvent preparation tank, piping, installation and other auxiliary components necessary for the operation of the plant.^86,87^

Operational expenditure (OpEx) was divided into cost of raw material (CRM), including fruit, solvents and other inputs; cost of utilities (CUT), mainly related to electricity consumption; cost of operating labor (COL); and cost of waste treatment (CWT).^86–88^

The cost of manufacturing (COM) was estimated according to a methodology widely used in technoeconomic studies of chemical and biotechnological processes.^87,89^ This parameter represents the overall cost associated with the operation of the plant and integrates the main economic components of the process.

The cost of extract production was determined by the ratio between the annual manufacturing cost and the annual extract production, according to eq 4.

4 $$C_{\mathrm{ex}}=\frac{\mathrm{COM}}{P_{\mathrm{year}}}$$

Where *C*_ex_ represents the cost of extract production (US$/kg); COM corresponds to the cost of manufacturing (US$/year); and *P*_year_ represents the annual extract production (kg/year).

This parameter was used to economically compare the different extraction systems evaluated.

### 4.8 Extraction Kinetics

For the extraction process that obtained the highest TPC content in the extract, the extraction kinetics study was conducted at temperatures of 20, 30, and 40 °C. To describe the kinetics of the process, the kinetic models listed in Table 13 were used.

**Table 13:** Kinetic Models Used to Fit the Experimental Data of TPC Extraction from *M*. *dubia*

kinetic model	equation^a^	parameters
Fick’s law	$C_{t}/C_{\max}=1-Ae^{\left(-B_{1}\cdot t\right)}$	*A* and *B*_1_
PSO	$C_{t}=t/\left[\left(\frac{1}{h}\right)+\left(\frac{t}{C_{\max}}\right)\right]$	*h* = *k*_2_ · *C*_max_^2^ and *k*_2_
partition	$C_{t}=\alpha \cdot \left[1-e^{\left(-a\cdot t\right)}\right]+\left[\beta -e^{\left(-b\cdot t\right)}\right]$	α, *a*, β and *b*
Weibull sorption	$C_{t}=A_{w}\cdot \left[1-e^{\left(-k_{w}\cdot t^{m}\right)}\right]$	A_w_, *k*_*w*_ (s^–1^) and *m*
Peleg’s sorption	$C_{t}=t/K_{1}+K_{2}\cdot t$	*K*_1_, *K*_2_ and *B*_0_ = 1/*K*_1_

a Where *C_t_* is the TPC concentration at any time (mg GAE/g); *C*_max_ is the TPC concentration at saturation (mg GAE/g); *A* is the pre-exponential constant of the model (dimensionless); *B*_1_ is the diffusion rate constant (s^–1^); *t* is time (s); *h* is the initial extraction rate (mg GAE.s/g); *k*_2_ is the second-order extraction rate constant (g/mg GAE.s); α is the partition parameter (mg GAE/g); *a* is the partition coefficient (s^–1^); β is the proportionality constant (mg GAE/g); *b* is the partition rate constant (s^–1^); *A*_w_ is the estimated maximum yield at infinite time (mg GAE/g); *k*_w_ is the constant related to the extraction rate (s^–1^); *m* is the distribution curve parameter (dimensionless); *K*_1_ is Peleg’s rate constant (mg GAE.s/g); *K*_2_ is Peleg’s capacity constant (g/mg GAE); and *B*_0_ is the extraction rate at the beginning of the process (mg GAE/g.s).

### 4.9 Mass Transfer Parameters

Through the pre-exponential coefficient (*A*) of Fick’s diffusive model, the coefficient *B*_1_ was obtained for spherical particles using eq 5.^90^

5 $$B_{1}=D_{\mathrm{eff}}\cdot \pi^{2}/r_{p}^{2}$$

Where *D*_eff_ is the effective diffusivity (m^2^/s) and *r*_p_ is the particle radius (m).

The relative magnitudes of the external and internal resistances to mass transfer (solid–liquid extraction) were determined by quantifying the *B*_*i*_, using eq 6.^90^

6 $$B_{i}=d_{p}{\cdot k}_{T}/D_{\mathrm{ext}}$$

Where *d*_p_ is the particle diameter (m); *k*_T_ is the mass transfer coefficient (m/s); *D*_ext_ is the external diffusion coefficient (m^2^/s).

The mass transfer coefficient was determined from the extraction kinetics using eq 7.^90^

7 $$\ln\left(C_{\max}/C_{\max}-C_{t}\right)=\left(A_{\mathrm{surf}}{\cdot k}_{T}/V_{\mathrm{so}}\right)\cdot t$$

Where *A*_surf_ is the surface area of the particle (m^2^) and *V*_so_ is the volume of solvent used (L).

### 4.10 Thermodynamic Parameters and Activation Energy

The thermodynamic parameters enthalpy (Δ*H*), entropy (Δ*S*), and Gibbs free energy (Δ*S*) of the TPC extraction process from the pulp of *M*. *dubia* were obtained using eq 8.^91^

8 $$\ln\left(k_{e}\right)=-\Delta H/R\cdot 1/T+\Delta S/R$$

Where *k*_e_ is the thermodynamic equilibrium constant; *R* is the universal gas constant (8.314 J/mol.K); *T* is the temperature (*K*).

The constant *k*_e_ was determined according to the literature, where the parameter *k*_e_ is the equilibrium constant for leaching, defined as the ratio between the amount of solute extracted and the amount of remaining unextracted solute, calculated by eq 9.^90^

9 $$k_{e}=C_{\max}/C_{\mathrm{ne}}$$

Where *C*_max_ is the concentration of extracted TPC at saturation and *C*_ne_ is the concentration of unextracted TPC (mg GAE/g).

The values of Δ*H* and Δ*S* were determined from the linear plot of ln­(*k*_e_) as a function of 1/*T* (Van’t Hoff equation), where the slope corresponds to −Δ*H*/*R* and the intercept to Δ*S*/*R*.

For the calculation of Δ*G* at different temperatures, eq 10 was used.^91^

10 ΔG=ΔH−ΔS·T

The activation energy (*E*_a_) was obtained using the Arrhenius eq (eq 11), assuming *D*_eff_ constant during the mass transfer process, which allows it to be used to describe the relationship between effective diffusivity and absolute temperature.^90^

11 $$\ln\left(D_{\mathrm{eff}}\right)=\ln\left(D_{0}\right)+\left(-E_{a}/R\cdot 1/T\right)$$

Where *D*_0_ is the pre-exponential factor, *R* is the universal gas constant (8.314 J/mol K), and *T* is the temperature (*K*).

### 4.11 Statistical Analyses

Analysis of variance (ANOVA),^92^ along with the Tukey test,^93^ using *p* ≤ 0.05, were performed to tabulate the obtained data.

## Supplementary Material



## Notes

Shear stress versus shear rate curves for ChCl:AcOH (Figure S1), ChCl:AcOH + H_2_O (Figure S2), and ChCl:AcOH + EtOH (Figure S3); scoring of the 12 AGREE principles for the extraction methods (Table S1) (PDF)
