# Supplementary material for: Extraction of Phenolic Compounds from the Pulp of Myrciaria dubia Using Eco-Friendly Solvents: Extraction Kinetics and Determination of Mass Transfer and Thermodynamic Parameters
Source: ACS Omega. 2026 Sep 18;11(38):56818–34. doi: 10.1021/acsomega.6c02599 (PMC13629028; doi:10.1021/acsomega.6c02599)
Supplement: Supplementary file 1 [file ao6c02599_si_001.pdf]

Title: Extraction of phenolic compounds from the pulp of *Myrciaria dubia* using eco-friendly solvents: extraction kinetics and determination of mass transfer and thermodynamic parameters

Authors: André Gomes Mesquita[1], Adriny dos Santos Miranda Lobato[1], Iane Valente Pires[1], Luiza H. M. Silva[1*] & Antonio M. C. Rodrigues[1]

Corresponding author: Luiza H. M. Silva – lhmeller@ufpa.br

Address: [1]Food Analysis Laboratory, Graduate Program in Food Science and Technology, Federal University of Pará, Rua Augusto Corrêa, 01, Belém, ZIP Code: 66075-110, Pará, Brazil.

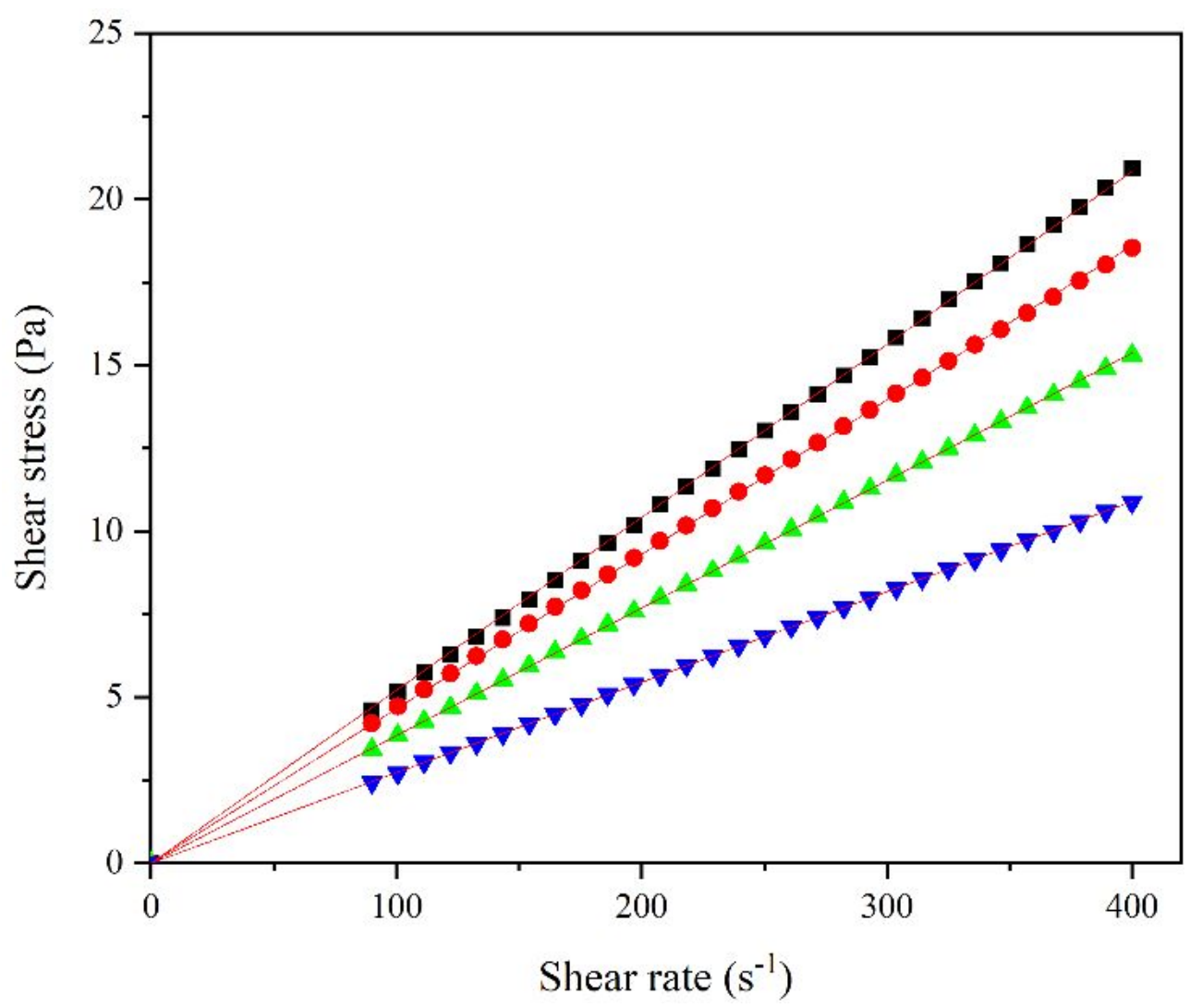

Figure S1. Shear stress as a function of shear rate for ChCl:AcOH at different temperatures. (■) 20 °C, (●) 25 °C, (▲) 30 °C, (▼) 40 °C, and (—) Newton model.

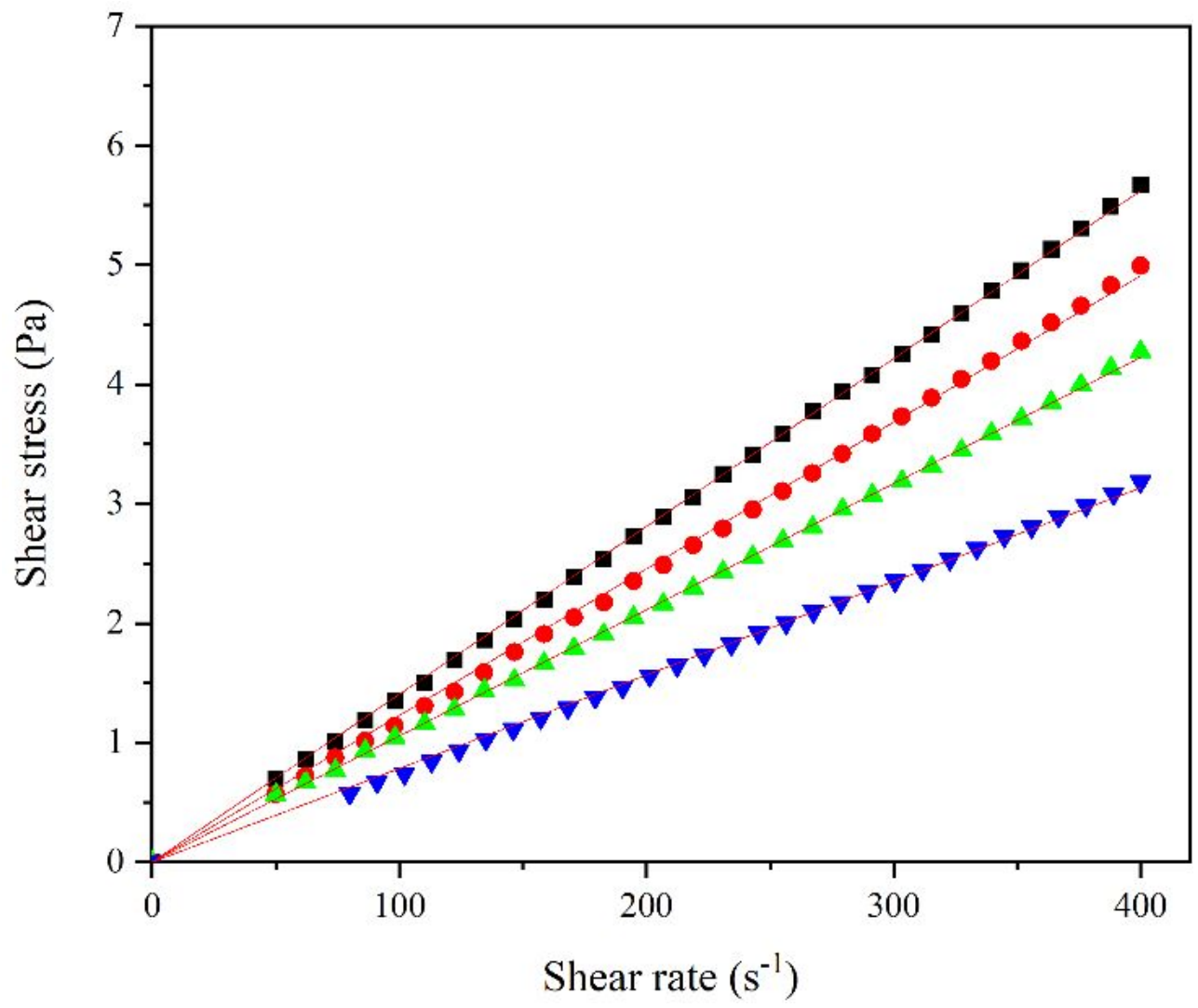

Figure S2. Shear stress as a function of shear rate for ChCl:AcOH + H_2O at different temperatures. (■) 20 °C, (●) 25 °C, (▲) 30 °C, (▼) 40 °C, and (—) Newton model.

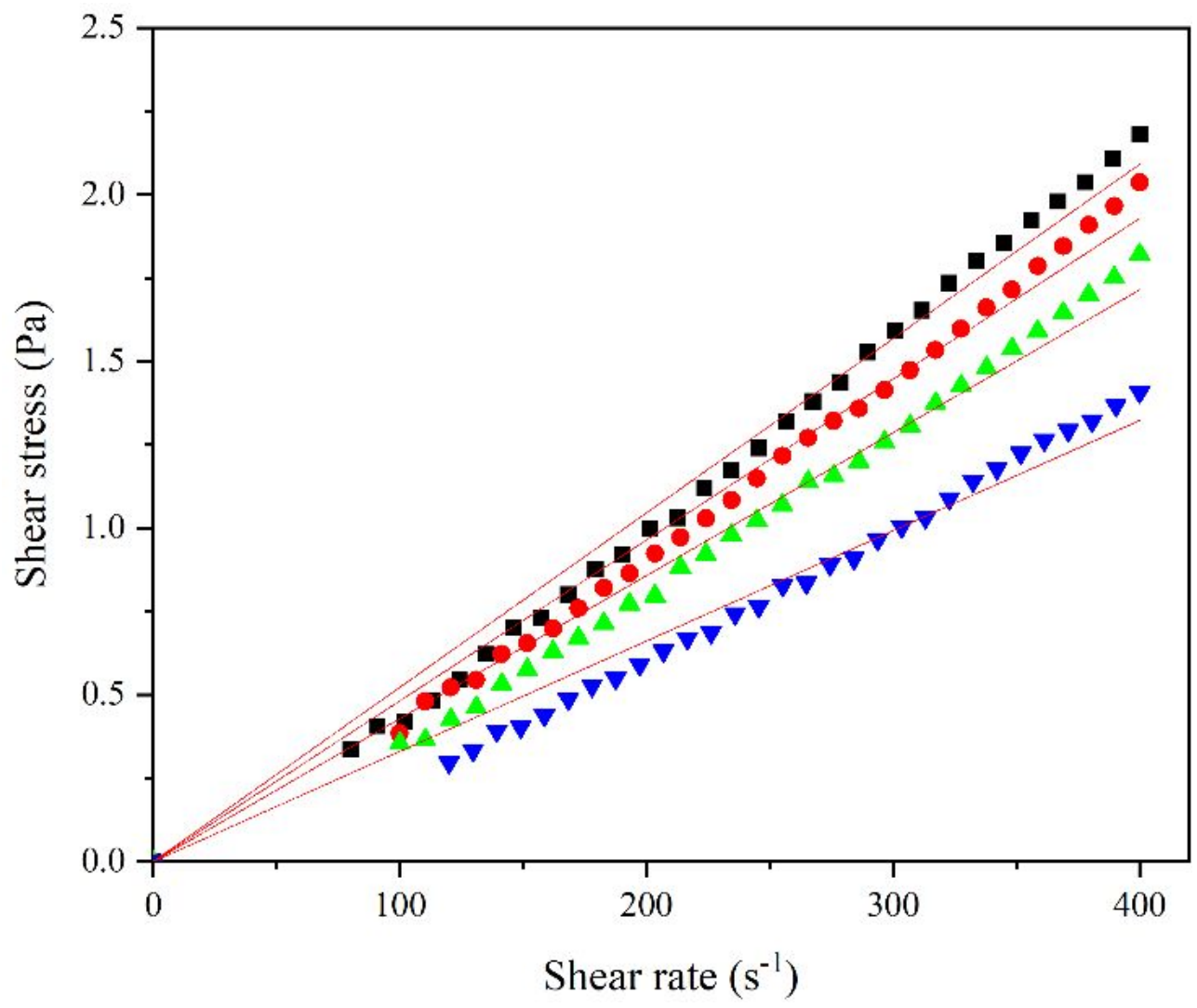

Figure S3. Shear stress as a function of shear rate for ChCl:AcOH at different temperatures. (■) 20 °C, (●) 25 °C, (▲) 30 °C, (▼) 40 °C, and (—) Newton model.

Table S1. Detailed scoring of the 12 AGREE principles for the extraction methods studied.

**AGREE principle**	**Solvent**			
	ChCl:AcOH	*ChCl:AcOH* + H_2O	*ChCl:AcOH* + EtOH	EtOH 70% (v/v)
Sample preparation	0.30	0.30	0.30	0.30
Sample size	0.65	0.65	0.65	0.65

Device positioning	0.00	0.00	0.00	0.00
Integration of steps	1.00	1.00	1.00	1.00
Automation	0.00	0.00	0.00	0.00
Derivatization	1.00	1.00	1.00	1.00
Waste	0.30	0.30	0.30	0.30
Multi-analyte	0.05	0.05	0.05	0.05
Energy consumption	1.00	1.00	1.00	1.00
Reagents source	1.00	1.00	1.00	1.00
Toxicity	1.00	1.00	1.00	0.11
Operator safety	1.00	1.00	1.00	0.80
**Final score**	**0.61**	**0.61**	**0.61**	**0.52**
